# Triggering Photoluminescence in High-Nuclear Silver Nanoclusters via Extra Silver Atom Incorporation

**DOI:** 10.1021/jacs.5c10289

**Published:** 2025-09-30

**Authors:** Aoi Akiyama, Sakiat Hossain, Sourav Biswas, Takafumi Shiraogawa, Pei Zhao, Mana Nakamoto, Daiji Ogata, Tokuhisa Kawawaki, Yoshiki Niihori, Junpei Yuasa, Masahiro Ehara, Yuichi Negishi

**Affiliations:** † Department of Applied Chemistry, 26413 Tokyo University of Science,1-3 Kagurazaka, Shinjuku-ku, Tokyo 162-8601, Japan; ‡ Research Institute for Science & Technology, Tokyo University of Science, Shinjuku-ku, Tokyo 162-8601, Japan; § Institute of Multidisciplinary Research for Advanced Materials, 13101 Tohoku University, Katahira, Sendai 980-8577, Japan; ∥ 88301 Institute for Molecular Science, 38, NishigoNaka, Myodaiji, Okazaki-shi, Aichi 444-8601, Japan

## Abstract

Photoluminescence (PL) in silver (Ag) nanoclusters (NCs) is intrinsically linked to their structural architecture, yet their low quantum yield at room-temperature hinders practical applications. Although various strategies have been explored to enhance the PL efficiency of Ag NCs, their effectiveness in high-nuclear Ag NCs remains largely uncertain. Here, we demonstrate a 77-fold enhancement in room-temperature PL quantum yield by modulating both radiative and nonradiative decay pathways in high-nuclear Ag NCs. A comparative study of two anion-templated Ag NCs, differing by a single Ag atom in the outermost shell, reveals that this substitution lowers structural symmetry, thereby increasing the radiative decay rate. This structural modification is facilitated by the alterations in ligands and their coordination environment, which simultaneously suppress atomic fluctuations and reduce the nonradiative decay component. Furthermore, theoretical investigations corroborate these findings, indicating that the incorporation of an additional Ag atom modifies the electronic distribution, thereby influencing the PL characteristics and ultimately altering the emission mechanism. These insights provide a deeper understanding of the structure–property relationship in high-nuclear Ag NCs and offer a rational strategy for enhancing their luminescence efficiency for potential applications.

## Graphical abstract

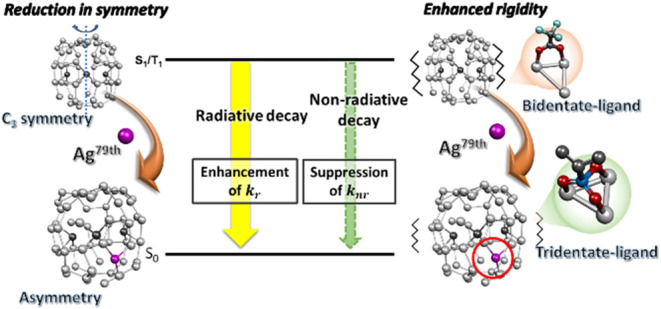

## Introduction

Over the past two decades, metal nanoclusters (NCs) have gained significant attention and experienced rapid advancements due to their well-defined structural architectures and correlated physicochemical properties.^1–3^ These characteristics set them apart from their larger counterparts, metal nanoparticles, which lack precise control over their structures. By taking advantage of their ultrasmall size, typically less than 3 nm in diameter, metal NCs exhibit discrete, quantized electronic states, leading to optical properties that are distinctly different from those observed in larger nanoscale materials.^4–7^ Among these optical properties, the molecular-like photoluminescence (PL) of metal NCs is particularly intriguing, as it opens up exciting possibilities for applications in sensing, bioimaging, and optoelectronics.^8–11^

NCs are typically composed of a well-defined metallic core encapsulated by a protective ligand/metal–ligand shell.^12–17^ Despite extensive research, the underlying mechanisms governing the PL of metal NCs remain a subject of debate.^8^ However, structural architecture has emerged as a crucial determinant of their optical properties.^18–21^ Among noble metal NCs, silver (Ag) NCs exhibit inherently brighter luminescence than their gold (Au) counterparts, primarily due to their more compact atomic arrangement and efficient charge transfer dynamics.^22–26^ However, the low intrinsic PL quantum yield (QY) of Ag NCs severely limits their practical applications, necessitating the development of effective strategies for enhancing their PL emission.^27^ In general, PL QY can be improved by either increasing the radiative decay rate or suppressing nonradiative decay pathways. Various strategies have been explored to achieve this goal, broadly classified into controlling by internal factors (e.g., ligand shell engineering, geometric rearrangements, heteroatom doping) and external factors (e.g., solvent polarity, temperature modulation, pH control, host–guest interactions).^28–33^ However, most reported approaches focus primarily on suppressing nonradiative decay, whereas radiative decay enhancement remains relatively less explored, primarily being influenced by core geometry modifications or heteroatom doping.^34,35^ While core geometry modifications can significantly impact PL properties, they often induce substantial structural distortions, leading to deviations from the intrinsic characteristics of the parent NC. Likewise, heteroatom doping can drastically alter the electronic structure, making direct comparisons between doped and undoped NCs difficult. These inherent limitations highlight the need for alternative strategies that boost luminescence while preserving the fundamental geometric and electronic integrity of the NCs.

In 2020, Yang et al. reported serendipitous synthesis of two distinct Ag NCs, [Ag_27_(S^*t*^Bu)_14_(S)_2_(CF_3_COO)_9_(DMAc)_4_]·DMAc (Ag_27_) and [Ag_28_(AdmS)_14_(S)_2_(CF_3_COO)_10_(H_2_O)_4_] (Ag_28_) (S^*t*^Bu: *tert*-butylthiolate; DMAc: dimethylacetamide; AdmS: adamantanethiolate), and investigated the role of a single Ag­(I) ion located within ligand shell of Ag_28_ in significantly improving PL while preserving the core structure.^36^ Unlike core modifications, the introduction of a single Ag atom in the ligand shell minimally perturbs the intrinsic electronic and geometric properties of the NC. Instead, it strengthens Ag–S and Ag–Ag interactions, enhancing the rigidity of the Ag–S framework. This increased structural rigidity ultimately suppresses the nonradiative decay pathways in the excited state by reducing dynamic atomic fluctuations, rather than directly influencing the radiative decay process. Although this approach enables studying the effect of two Ag NCs with preserved core geometry, its use remains limited due to the synthetic complexity, hindering precise single-atom modifications in high-nuclear NCs. Notably, there are no reported investigations in the literature that controlled the growth of high-nuclear Ag NCs in such a way and studied their impact on PL modulation without compromising structural integrity. However, the methodologies reported by Yang et al.^36^ paved the way for further exploration of alternative strategies capable of directly enhancing the radiative decay process, thereby facilitating the development of highly emissive Ag NCs with superior optoelectronic functionalities.^32^

To gain deeper insight into this complex system, here we synthesized two high-nuclearity anion-templated Ag NCs; [SO_4_@Ag_78_S_15_(CpS)_27_(CF_3_COO)_18_]^+^: Ag_78_ NC (CpS: cyclopentanethiolate), [SO_4_@Ag_79_S_15_(^*i*^PrS)_28_(^*i*^PrSO_3_)_15_(CF_3_COO)_4_]: Ag_79_ NC (^*i*^PrS: iso-propyl thiolate), and compared their emission characteristics. The key distinction between these two NCs lies in the incorporation of a single additional Ag atom in the shell of Ag_79_ NC, achieved through modifications in the surface-protecting ligand system, while the core structure remained largely consistent. The modified ligand system, especially in situ generated ^*i*^PrSO_3_^–^, led to an overall coordination environment enabling the incorporation of an extra Ag atom into a void space generated within the cluster framework of Ag_79_. Interestingly, such incorporation of the extra Ag atom in Ag_79_ NC reduces in the overall cluster symmetry compared to Ag_78_ NC. This symmetry reduction played a crucial role in triggering an enhanced radiative decay process, as supported by theoretical calculations. Additionally, the modification in the ligand environment and the presence of the extra Ag atom effectively restricted the structural dynamics of the cluster by shortening Ag–S and Ag–Ag distances. This structural rigidity significantly suppressed nonradiative decay pathways, thereby improving the overall emission efficiency. As a result of these synergistic factors—the symmetry reduction leading to enhanced radiative decay and the restricted motion reducing nonradiative losses—the incorporation of a single Ag atom in the shell of Ag_79_ NC remarkably boosted the room-temperature PL QY of Ag_78_ NC by an impressive 77-fold.

## Results and Discussion

### Synthesis of Ag_78_ and Ag_79_ NCs: Influence of Ag­(I) Precursors and Cu­(II) Cations

In NC synthesis, ligands play a fundamental role in directing the assembly of metal ions, with metal–ligand interactions significantly impacting the nuclearity and structural framework of the resulting NC.^37–40^ However, in this study, two [SO_4_]^2–^ -templated Ag NCs, Ag_79_ and Ag_78_, with very similar metal framework structures were synthesized using a similar one-pot synthesis approach, despite a significant differences in NC’s surface-protecting ligand systems (only CpS^–^ and CF_3_COO^–^ in Ag_78_ vs ^*i*^PrS^–^, ^*i*^PrSO_3_^–^ and CF_3_COO^–^ in Ag_79_). This highlights the critical role of the choice of metal precursor, reactant ratios, in situ ligand formation, and reaction conditions in the synthesis of atomically precise NCs.

In a typical synthesis, Ag_78_ NCs were obtained by treating a [CpSAg]*_n_* complex with CF_3_COOAg dissolved in a methanol/dichloromethane mixture in the presence of sulfate anions (Figure S1). Here, soluble Ag­(I) salt, CF_3_COOAg, helps depolymerize the polymeric Ag­(I) precursor [CpSAg]*_n_* and in situ generation of [S]^2–^ anions, promoting assembly of NCs by incorporating [S]^2–^ and CF_3_COO^–^ anions. Brown, block-shaped crystals are formed on keeping the resulting yellow solution over 2 weeks in the dark. In contrast, the synthesis of Ag_79_ NCs involved a modified approach, where a solution mixture of CF_3_COOAg and Cu­(NO_3_)_2_ was directly treated with ^*i*^PrSH in an acetone/acetonitrile medium (Figure S1). Up on addition of ^*i*^PrSH, blue color of the solution changes to faint blue and again back to blue. The solution was allowed to evaporate very slowly (ensured by making a very small hole on the plastic cap of the crystallization vial) under ambient conditions, yielding dark brown block-shaped crystals after one month (Figure S1). A portion of added thiol reacted with Ag­(I) of CF_3_COOAg to form assembly made of Ag–S-^*i*^Pr and [S]^2–^. Simultaneously another fraction of the added ^*i*^PrSH reacts with Cu­(II), facilitating thiol oxidation via highly reactive thiyl radicals that generate ^*i*^PrSO_3_H.^41^ Subsequent oxidation of ^*i*^PrSO_3_H induces S–C bond cleavage, producing [SO_4_]^2–^ anions, which act as templating anion for the formation of Ag_79_ NC. This sequence explains the observed color change of the solution from blue (due to Cu­(II) ions) to faint blue (as part of blue colored Cu­(II) ions is reduced by added ^*i*^PrS^–^ to colorless Cu­(I)) and finally to blue as Cu­(I) reoxidized back to Cu­(II) by atmospheric oxygen. We believe this is the reason Cu­(II) was not incorporated in the final Ag_79_ NC. Thus, Cu­(II) plays a key role in generating optimum amount of [SO_4_]^2–^ anions, enabling the [SO_4_]^2–^ templated synthesis of Ag_79_ NCs.

### Unveiling Geometric Insights through Structural Analysis

Single-crystal X-ray diffraction (SCXRD) analysis reveals that Ag_78_ NC crystallizes in a trigonal crystal system with a space group of *P-*3, whereas Ag_79_ NC adopts a monoclinic crystal system with a space group of *C*12*/c*1 (Tables S1 and S2). The crystallographic data confirm that the overall composition of both NCs aligns with their respective molecular formulas mentioned previously and structurally depicted in Figure 1. Both Ag_78_ and Ag_79_ NCs incorporate [SO_4_]^2–^ as a templating anion which plays a pivotal role in guiding the arrangement of Ag­(I) atoms (Figures 1 and 2). This templating effect facilitates the formation of a structurally similar Ag_18_ core in both NCs, stabilized primarily through argentophilic interactions (Figure 2).^42^ In the case of Ag_78_ NC, [SO_4_]^2–^ anion is externally introduced into the reaction medium via the direct addition of sodium sulfate. This ensures the immediate availability of sulfate ions, enabling their direct involvement in coordination-driven self-assembly of Ag­(I) atoms. Control experiments without added sulfate fail to yield the desired product, highlighting the crucial role of preintroduced sulfate in nucleation and growth, thereby promoting synthesis and subsequent crystallization of Ag_78_ NC. In contrast, the Ag_79_ NC follows a different pathway, where the templating sulfate anion is not externally supplied but is instead generated in situ throughout the reaction.^43^ This in situ formation of [SO_4_]^2–^ is governed by a multistep oxidative process catalyzed by Cu­(II) species as described previously.^41,44^

**Figure 1:**
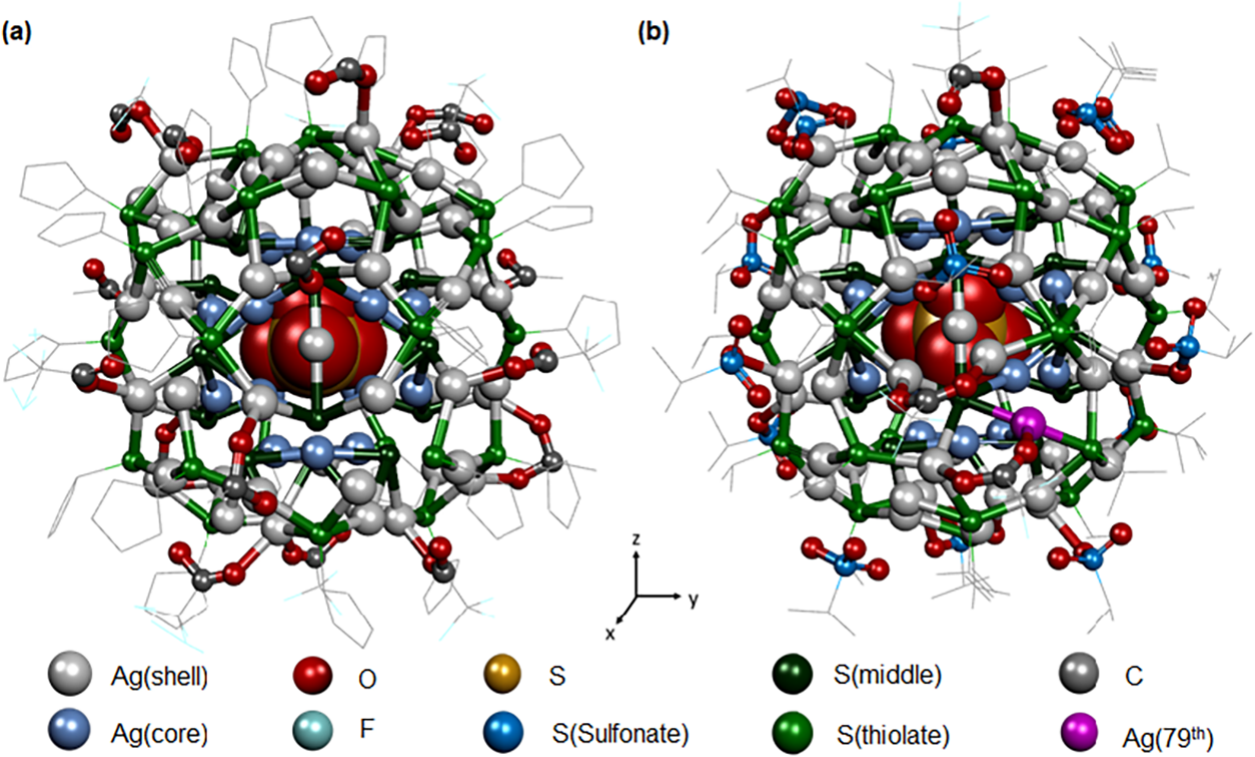
Structural architectures of anion-templated (a) Ag_78_ and (b) Ag_79_ NCs. Hydrogen atoms are omitted for clarity.

**Figure 2:**
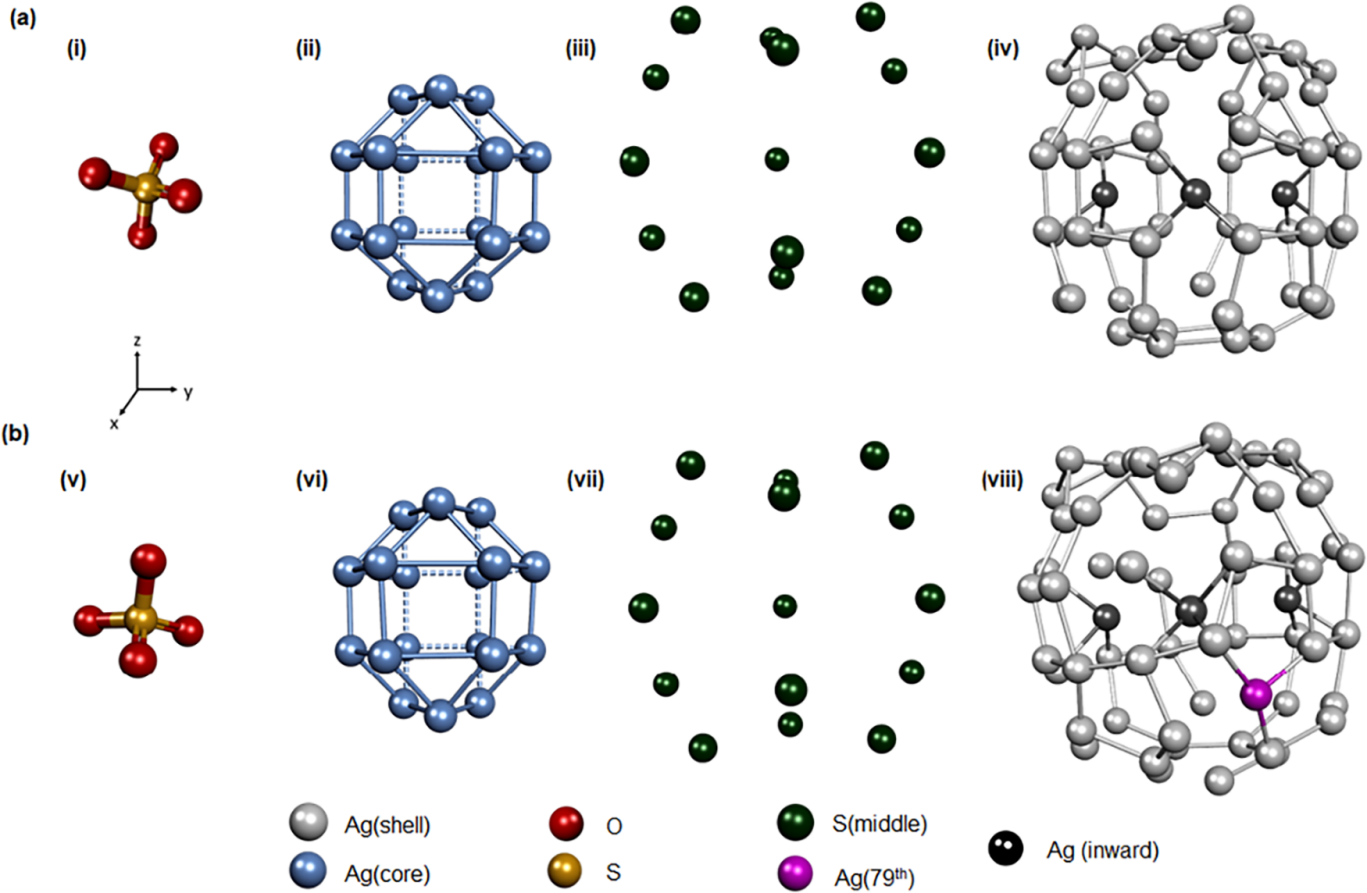
Detailed structural anatomy of (a) Ag_78_ NC where (i) anion template, (ii) geometry of 18 Ag­(I) atoms that encapsulate the template anion and form the metal core, (iii) metal core is encapsulated by 15 [S]^2–^ ligands, and (iv) 60 Ag­(I) atoms forming the outer shell of the NC. Detailed structural anatomy of (b) Ag_79_ NC where (v) anion template, (vi) geometry of 18 Ag­(I) atoms that encapsulate the template anion and form the metal core, (vii) metal core is encapsulated by 15 [S]^2–^ ligands, and (viii) sixty-one Ag­(I) atoms forming the outer shell of the NC.

Despite the distinct pathways involved in the formation of templating oxoanions, both Ag_78_ and Ag_79_ NCs exhibit a strikingly similar core geometrical arrangement, consisting of 18 Ag­(I) atoms. In both cases, the Ag­(I) atoms are arranged in an elongated triangular orthobicupola geometry, where their connections form eight triangles and 12 rectangles (Figure 2). More simply, the structure consists of two triangular cupolas at opposite ends, interconnected by six equatorial rectangular units that act as bridges. These equatorial rectangular units play a crucial role in maintaining the overall rigidity of the NCs, ensuring structural integrity under various conditions. A detailed comparison of the Ag–Ag bond lengths reveals subtle structural differences in the average argentophilic interaction between the two Ag_18_ cores. In Ag_78_ NC, the average Ag–Ag bond length within the Ag_18_ is 3.192 ± 0.051 Å, while in Ag_79_ NC, it is slightly shorter at 3.051 ± 0.013 Å. This slight contraction in the Ag_18_ core arrangement indicates subtle structural adjustments, resulting in a minor distortion, likely driven by variations in the coordination of the templating anion with the Ag atoms. In Ag_79_ NC, the templating anion adopts a μ_5_ coordination mode with adjacent Ag­(I) atoms, resulting in an average Ag–O bond length of 2.611 ± 0.026 Å. In contrast, in Ag_78_ NC, the anion is coordinated in a μ_3_ mode, with an Ag–O bond length of 2.635 Å.

Further evaluation reveals that these Ag­(I)-based cationic cores are intricately encapsulated by a sulfide [S]^2–^ anionic shell, which consists of 15 [S]^2–^ ligands (Figure 2). This type of encapsulation plays a crucial role in stabilizing the overall structure of Ag NCs by providing a well-defined coordination environment that mitigates electronic repulsions and enhances structural integrity. Although these [S]^2–^ anions are not intentionally introduced during synthesis in either case, they are generated in situ through the dissociation of respective thiols.^45–48^ However, the spatial distribution of the 15 [S]^2–^ ligands follows a well-defined pattern where 12 [S]^2–^ ligands are positioned on each facet of the triangular cupolas, effectively capping and stabilizing the terminal sections of the cluster core (Figure S2). The remaining three [S]^2–^ ligands are strategically located on alternating rectangular facets in the equatorial region of the core (Figure S2).

Further analysis of the [S]^2–^ ligand distribution reveals that the structure can be divided into five distinct layers, each composed of three [S]^2–^ anions (Figure S3). This layered organization influences the overall bonding characteristics and contributes to the subtle variations observed in the Ag–S bond lengths. The Ag–S bond lengths exhibit slight positional variations within the NCs, which can be attributed to differences in local coordination environments and minor geometric distortions. Specifically, in the Ag_78_ NC, the Ag–S bond lengths at the triangular cupola facets range from 2.454 to 3.048 Å, while the bond lengths at the bridging rectangular facets are slightly shorter, varying between 2.513 and 2.557 Å. These values indicate a relatively uniform coordination environment with minimal distortions. However, in the Ag_79_ NC, the Ag–S bond lengths exhibit a slightly broader range due to subtle geometric distortions in the Ag_18_ core. Specifically, the Ag–S bond lengths at the triangular cupola facets vary between 2.409 Å and 3.027 Å, whereas at the bridging rectangular facets, the bond lengths range from 2.455 to 2.815 Å.

In both Ag_78_ and Ag_79_ NCs, the [S]^2–^ layer is encapsulated by an outermost cationic shell, comprising 60 Ag­(I) atoms in Ag_78_ NC and sixty-one Ag­(I) atoms in Ag_79_ NC (Figure 2). This difference in the number of Ag­(I) atoms in the outermost shell leads to a distinct variation in their structural architecture. However, in both cases, the outermost cationic shell is structured into two distinct layers of Ag­(I) atoms, contributing to the stability and integrity of each NC. Within the middle section of the outer shell, three Ag­(I) atoms exhibit a slight inward displacement beneath the surface (Figure 2). This shift results from the alternating face-capping arrangement of [S]^2–^ anions at the equatorial rectangular facets of the Ag_18_ core (Figure S4). In regions where these anions are absent, the Ag­(I) atoms experience a noticeable inward shift toward the core through the direct interaction with the core. So, the remaining layer of the outermost cationic shell contains fifty-seven Ag­(I) atoms for Ag_78_ NC and fifty-eight Ag­(I) atoms for Ag_79_ NC (Figure S5). Although the arrangement of these Ag­(I) atoms does not follow any regular geometric pattern, their spatial arrangement creates another three-layered system (horizontally) for both NCs (Figure S6). As it has already been observed that the inner cores exhibit almost regular geometric structures with a precise arrangement of Ag­(I) atoms but the outer shells experience an uneven distribution of Ag­(I) atoms outside of the triangular cupolas. For Ag_78_ NC, one side of the triangular cupolas encapsulated by 18 Ag­(I) atoms and the other side is encapsulated by 15 Ag­(I) atoms (Figure S6). On the other hand, for Ag_79_ NC, the triangular cupolas are encapsulated by 18 and 16 Ag­(I) atoms respectively (Figure S6). However, the edges of the bridging equatorial rectangles of the core in both NCs are protected by similar 24 Ag­(I) atoms from the outside through Ag–S coordination (Figure S6). Thus, the addition of a single atom to one layer of the outer cationic shell leads to significant overall differences. This unexpected incorporation of a single Ag­(I) atom on one side of the NC disrupts the inherent *C*_*3*_ symmetry observed in Ag_78_ NC (Figure S7).

The outermost layer of the cationic shell in both Ag_78_ and Ag_79_ NCs is coordinated with a diverse set of surface-protecting ligands, which play a crucial role in dictating the structural rigidity and interatomic interactions (Figure 3). In Ag_78_ NC, the ligand shell is composed of twenty-seven monodentate CpS^–^ ligands, which coordinate via μ_3_ and μ_4_ bridging modes, establishing an average Ag–S bond length of 1.815 ± 0.014 Å (Figures 3a andS8a). Additionally, 18 bidentate CF_3_COO^–^ ligands are attached to the surface through μ_3_ and μ_2_ coordination modes, with an average Ag–O bond distance of 2.421 ± 0.027 Å (Figures 3a andS8b). On the other hand, in Ag_79_ NC, the ligand environment exhibits notable differences (Figure 3b). The NC is protected by twenty-eight monodentate ^*i*^PrS^–^ ligands, which are attached through μ_4_ and μ_3_ coordination modes, forming Ag–S bonds with an average length of 2.534 ± 0.028 Å (Figures 3b andS9). In contrast to Ag_78_, Ag_79_ features only four bidentate CF_3_COO^–^ ligands, which coordinate exclusively via the μ_2_ mode, with a slightly shorter average Ag–O bond length of 2.335 ± 0.025 Å (Figures 3b andS9). Moreover, Ag_79_ incorporates an additional set of tridentate ^*i*^PrSO_3_^–^ ligands, which are formed in situ through Cu­(II)-catalyzed oxidation of ^*i*^PrSH. These tridentate ligands exhibit a broader range of coordination, interacting through μ_3_, μ_4_, and μ_5_ modes, with an average Ag–O bond length of 2.439 ± 0.019 Å (Figures 3b andS9).

**Figure 3:**
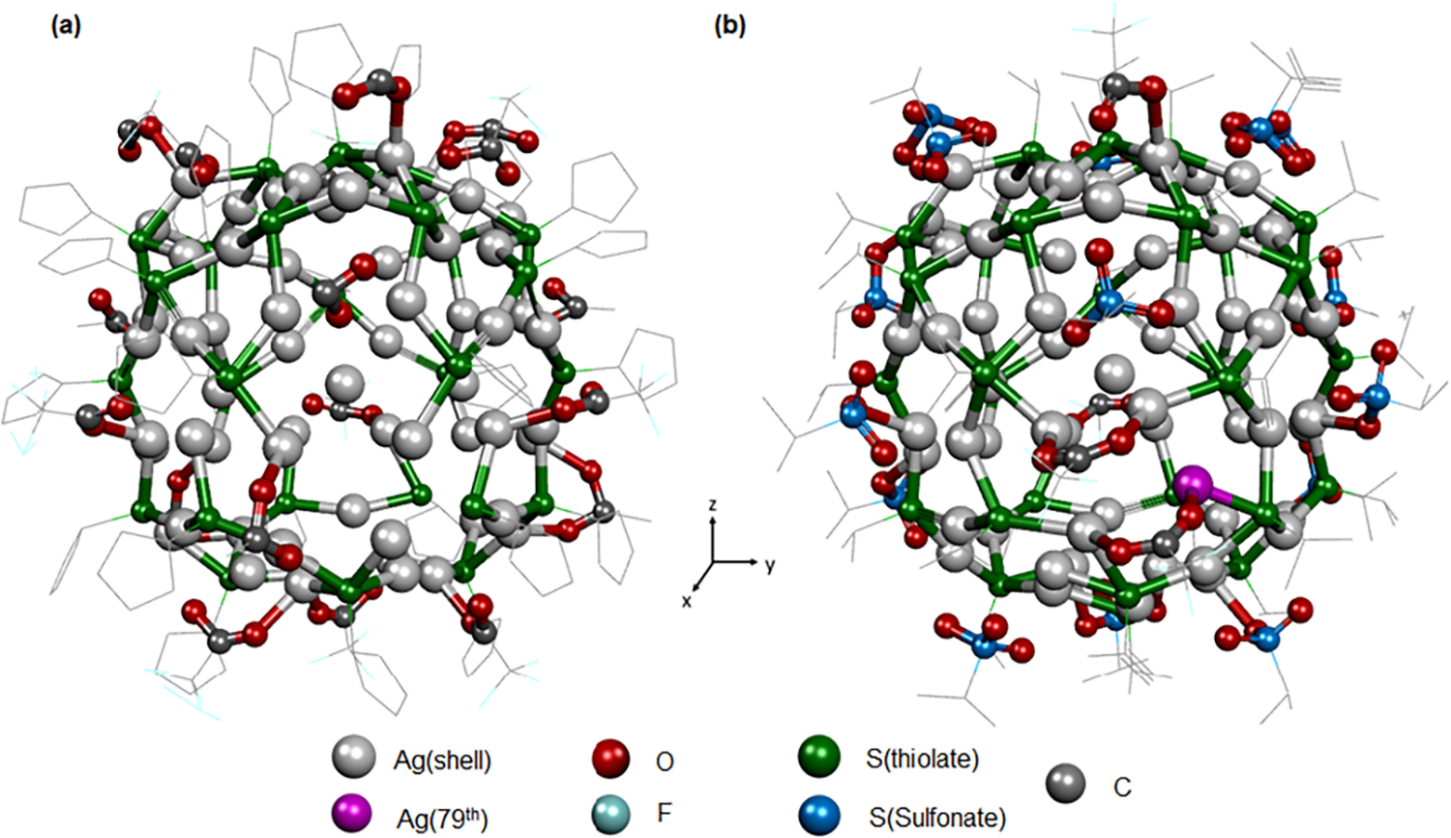
Metal atoms in the outer shell and the associated surface-protecting ligands in (a) Ag_78_ and (b) Ag_79_ NCs. Hydrogen atoms are omitted for clarity.

The presence of these distinct ligand systems leads to a clear structural divergence between the two NCs in the outermost layer. Notably, Ag_78_ lacks a tridentate ligand system, whereas Ag_79_ incorporates 15 bulkier ^*i*^PrSO_3_^–^ ligands by replacing 14 bidentate CF_3_COO^–^ ligands along with one additional thiolate ligand. This ligand substitution significantly alters the surface environment, introducing greater steric constraints through its tridentate coordination mode, which increases the number of Ag–O bonds. The enhanced steric hindrance imposed by the tridentate ligands increases surface rigidity, affecting the Ag–Ag bond interactions more prominently than in the bidentate ligand system. Notably, the Ag–Ag bond length at the coordinating sites of the tridentate ligands in Ag_79_ is considerably shorter (3.208 ± 0.123 Å) compared to the corresponding bond length at the bidentate ligand coordinating site in Ag_78_ NC (3.565 ± 0.142 Å) (Figure S10). This decrease in bond length underscores the significant influence of the ligand system on argentophilic interactions and its crucial role in shaping the structural framework of the NCs. The alteration in bond length creates additional space, facilitating the insertion of an extra Ag­(I) atom in the outermost layer. As a result, these structural modifications further influence the overall dynamics of the NC, ultimately leading to a contraction in the Ag–Ag bond length within the outermost layer of the cationic shell. Specifically, the average Ag–Ag bond length decreases from 3.147 ± 0.043 Å in Ag_78_ to 3.054 ± 0.017 Å in Ag_79_, further emphasizing the structural impact of ligand substitution. Additionally, electrostatic attraction and weak hydrogen bonding (C–H···F) between individual NCs contribute to the stabilization of the crystal packing, reinforcing the overall stability and organization of the system (Figure S11).

To theoretically analyze the geometric structures of these two NCs, density functional theory (DFT) calculations were performed for the real systems without simplifying the ligands. Details of the procedures including the dependence on the chosen DFT functional, are provided in the Supporting Information. The structural parameters along with natural population analysis (NPA) charges, and Wiberg bond order (WBO) indices of these clusters are summarized in Tables S3–S6. DFT calculations reveal that the local minimum structure of Ag_78_ NC exhibits an almost *C*_*3*_ symmetry whereas Ag_79_ NC shows reduced symmetry, consistent with the SCXRD analysis. The root-mean-square deviation (RMSD) of the calculated Ag NC structure parameters from those obtained via SCXRD, based on the Kabsch algorithm, was 0.46 Å for both NCs (Figure S12). This indicates that the optimized geometries closely resemble the experimentally determined structures without significant deviations. The NPA revealed that the charge of the templating anion varied depending on the basis sets used (Tables S3 and S4), although such charges indicate that the interaction between the templating anion and Ag atoms is predominantly electrostatic. Additionally, the calculated shortest Ag–O distances ranged from 2.755 to 2.430 Å for Ag_78_ NC and from 2.689 to 2.592 Å for Ag_79_ NC, depending on the position of the templating anion within the core. The WBOs for these short Ag–O distances were 0.037–0.036 for Ag_78_ NC and 0.028–0.025 for Ag_79_ NC, indicating weak covalent interactions between Ag and O in both cases. The interaction of the Ag core with SO_4_ anion was also examined by the energy decomposition analysis-natural orbitals for chemical valence (EDA-NOCV) (Tables S7 and S8). These values are slightly larger than those of halide and chalcogenide anion-templated Ag NCs.^49^ These high energies indicated that the electrostatic interaction is dominant, while the orbital interaction also exists.

### Exploring Molecular Insights through Characterization

In the positive mode electrospray ionization mass spectrometry (ESI-MS) spectrum, the Ag_78_ NC displays multiple peaks in the *m*/*z* range of approximately 6500 to 8000, corresponding to various fragment species. Among them, two prominent molecular ion peaks are identified at *m*/*z* 6827.6 and 6931.0 designated as (i) and (ii) (Figure S13, Table S9). The multiple fragmentation pattern suggests that this NC undergoes ligand desorption under the ionization conditions. Interestingly, a previously reported Ag_78_ NC with a similar structural framework displayed a comparable fragmentation behavior in its ESI-MS spectrum.^50^ This observation strongly indicates that the surface ligands of Ag_78_ NC are relatively labile and can be easily removed during the ESI-MS experimental process. In contrast, the ESI-MS spectrum of Ag_79_ NC reveals a distinct fragmentation pattern, with only three distinguishable peaks centered at *m*/*z* 6482.3, 6524.2, 6640.3 which are assigned to (iii–v) respectively (Figure S14). The presence of fewer fragmentation peaks suggests that Ag_79_ NC possesses a more robust structural framework compared to Ag_78_ NC. The assigned formulas corresponding to each peak for both NCs are systematically summarized in Table S9. Although the overall charge state of the structurally rigid Ag_79_ NC in its native form is neutral, under ionization conditions, the NC still experiences partial loss of its surface-associated ligands. However, due to the presence of a variety of coordinating ligands, the desorption tendency varies among them. A detailed analysis of the fragmentation pattern reveals that the CF_3_COO^–^ ligand is more prone to desorption compared to other ligands. This variation in ligand desorption behavior is attributed to the nature of the coordinating ligands and their relative binding affinities. However, a concentration-dependent fragmentation behavior is also observed in Ag_79_ NC during ESI-MS measurements, wherein lower concentrations result in fragmentation patterns that shift toward higher *m*/*z* values (Figure S15). Based on the principles of the hard and soft acids and bases theory, oxygen-coordinating ligands exhibit weaker coordination to the Ag compared to thiolate ligands, which primarily govern the desorption process here. However, between the two oxygen-coordinating ligands, the ^*i*^PrSO_3_^–^ ligand, due to its tridentate coordination mode, requires relatively higher energy to be desorbed from the surface than the bidentate CF_3_COO^–^. Consequently, when the concentration of CF_3_COO^–^ ligands in the solvent are lower, ligand exchange reactions proceed more readily, shifting the equilibrium toward preferential desorption of CF_3_COO^–^ ligands while forming ^*i*^PrSO_3_^–^ coordination. This equilibrium shift can be represented by the equation: ([SO_4_@Ag_79_S_15_(^*i*^PrS)_28_(^*i*^PrSO_3_)_15_(CF_3_COO)_4_]⇌[SO_4_@Ag_79_S_15_(^*i*^PrS)_28_(^*i*^PrSO_3_)_15+*x*_(CF_3_COO)_4–x_]), which results ESI mass spectrum shifts to the higher *m*/*z* region (Figure S16). However, the presence of the functional groups outside the NCs in ambient condition is confirmed by the FT-IR measurement (Figure S17). Further, the purity of these NCs in the bulk phase is characterized by X-ray photoelectron spectroscopy (XPS) measurement. The survey spectrum of both NCs confirms the absence of any contamination by any other elements, despite the incorporation of other metal salts during the course of the reactions (Figure S18). The high-resolution binding-energy spectra confirm the presence of Ag 3d peaks in both of these NCs, which indicates the sole presence of Ag­(I) in these NCs (Figure S19). However, there is a difference in the binding-energy spectrum of S 2p between these two NC. The deconvoluted peak ∼162 eV region for Ag_78_ NC confirms the presence of thiolate and [S]^2–^ ligands, which is also consistent with the Ag_79_ NC. But Ag_79_ NC contains an additional peak ∼168 eV, which corresponds to the ^*i*^PrSO_3_^–^ ligand (Figure S20).

### Probing the Connection between Optical Behavior and Structural Robustness

The ultraviolet-Visible (UV–vis) absorption spectra of these NCs were recorded in a dichloromethane solution. Both NCs exhibit three distinct absorption peaks at 390, 450, and 550–600 nm, indicating similar electronic transitions (Figure 4). The solid-state diffuse reflectance spectra also show a similar pattern, suggesting the structural stability of these NCs in the absence of solution (Figure 4). Furthermore, their long-term stability was evaluated by monitoring time-dependent changes in their optical absorption spectra in solution (Figure S21). Based on the peak positions in the time-dependent spectra for both NCs, it is estimated that even after 96 h, these NCs are mostly stable in nature. However, the emergence of insoluble components has begun in both NCs, leading to an increased baseline in the long-wavelength region. Notably, the onset time frame of these insoluble components differs between the two NCs, reflecting their varying stabilities in the solution medium. The appearance of such components for Ag_78_ NC began after 60 h, whereas for Ag_79_ NC it appeared after 84 h. Such a change is might be associated with the change in the structural rigidity and the alteration in the argentophilic interaction which linked to the robust structural skeleton. The photostability of both nanoclusters was further evaluated under ambient light conditions over a 24 h period (Figure S22).

**Figure 4:**
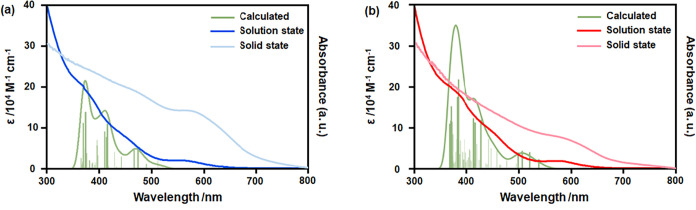
Solution-state, solid-state and theoretical UV–vis absorbance spectra of (a) Ag_78_ and (b) Ag_79_ NCs.

To gain deeper insight into the electronic transitions associated with the optical properties of these NCs, time-dependent DFT (TD-DFT) calculations were performed for both Ag_78_ and Ag_79_ NCs. The simulated absorption spectra exhibited fair agreement with the experimentally observed spectra (DFT functional dependence of the absorption spectra was also analyzed for Ag_79_ NC Figure S23), capturing all the three prominent absorption features for both of these NCs (Figure 4). The molecular orbitals (MOs) relevant to these absorption peaks are illustrated in Figures S24 and S25. A comparative analysis of the calculated absorption spectra reveals that the peaks in Ag_78_ NC are slightly blue-shifted relative to those in Ag_79_, with weaker oscillator strengths (Tables S10 and S11). This distinct absorption behavior of Ag_78_ NC can be attributed to the *C*_*3*_ symmetry of its structural framework, which influences the electronic distribution within the NC. For Ag_79_ NC, the lowest-energy absorption peak corresponds to excited states calculated within the 499–538 nm range. These peaks are attributed to the transitions from HOMO–4 −highest occupied molecular orbital (HOMO) to lowest unoccupied molecular orbital (LUMO) (Table S11). Based on the orbital decomposition analysis (Tables S12 and S13), these transitions are predominantly characterized as ligand-to-metal charge transfer (LMCT) from S-based ligand orbitals to Ag orbitals, along with a local excitation (LE) component within the NC. The second and third absorption peaks of Ag_79_ NC are also attributed to a combination of LMCT and LE transitions. Specifically, the second peak originates from excited states calculated within the 414–428 nm range, while the third peak corresponds to transitions occurring in the 370–385 nm region. Notably, these transitions primarily involve electron density redistribution toward the LUMO, as confirmed by the analysis of electron density differences between the ground and excited states (Figure S26), which further supports the dominance of LMCT character in these absorptions. Similarly, in Ag_78_ NC, the key electronic transitions involve molecular orbitals predominantly localized on S atoms transitioning to orbitals localized on Ag atoms, leading to absorption features primarily categorized as LMCT and LE, akin to Ag_79_ NC. However, the nature of these transitions exhibits a fundamental difference. As seen from the relevant MOs and orbital decomposition analysis (Figure S24, Table S12) and the electron density differences between the ground and excited states (Figure S27), the transitions in Ag_78_ NC appear more delocalized across the cluster compared to the relatively localized nature observed in Ag_79_ NC. Although the presence of an additional Ag and S atom in Ag_79_ NC has only a minimal impact on its absorption spectrum compared to Ag_78_ NC, the structural distortions caused by these extra atoms result in significant modifications to the electron density distribution. This highlights the crucial role of NC architecture in fine-tuning electronic and optical properties.

Upon irradiation under a UV light source (365 nm), Ag_79_ NC in dichloromethane exhibits a detectable red PL emission at room temperature (RT) (Figure 5a inset). The PL spectrum upon excitation at 450 nm reveals a well-defined peak centered at 710 nm for Ag_79_ NC, indicating the presence of characteristic electronic transitions within the NC (Figure 5a). In contrast, under identical experimental conditions, Ag_78_ NC displays only a very weak PL with an emission maximum at 720 nm, indicating a significant difference in their emissive properties (Figure 5a). The PL QY of Ag_78_ NC and Ag_79_ NC were determined using the relative method with oxazine-1 as the reference material, yielding values of 0.001 and 0.0773, respectively (Table S14).^21^ Interestingly, Ag_79_ NC exhibits a significantly higher molar absorption coefficient (3.48 × 10^4^ M^–1^cm^–1^) in dichloromethane at 452 nm, compared to Ag_78_ NC (1.91 × 10^4^ M^–1^cm^–1^), adding complexity to the interpretation of their photophysical behavior (Figure S28). Despite this, the solid-state PL spectra of both NCs closely resemble their solution-state counterparts, indicating this enhanced PL emission behavior can be attributed to the presence of an additional Ag­(I) atom and modifications in the ligand environment, which play a crucial role in stabilizing the emissive excited states, thereby leading to higher quantum efficiency (Figure 5b). However, both NCs exhibited slight red shifts in their solid-state emission maxima, likely due to reduced solvent interactions. A further significant enhancement in the emission intensities of these NCs is observed as the measurement temperature decreases (Figure 5c). At 77 K, the Ag_78_ NC exhibits an emission intensity that is 223 times higher than its RT value, while Ag_79_ NC shows a 10-fold increase (Table S15). These substantial increases in emission intensities by decreasing temperature are generally attributed to the suppression of nonradiative decay channels.^15^ Additionally, this observation suggests that nonradiative relaxation, driven by the vibrations of the ligands, plays a significant role in the behavior of these NCs. Interestingly, a blue shift in the emission maximum is also observed at lower temperatures. To better understand this shift, temperature-dependent measurements were conducted between 293 and 173 K, with 10 K intervals for Ag_79_ NC. The results consistently show a blue shift in the emission maximum as the temperature decreases (Figure S29). This behavior indicates that the molecular vibrations of the ligands become increasingly restricted at lower temperatures, thereby suppressing the excitation transitions originating from the metal center.^15^

**Figure 5:**
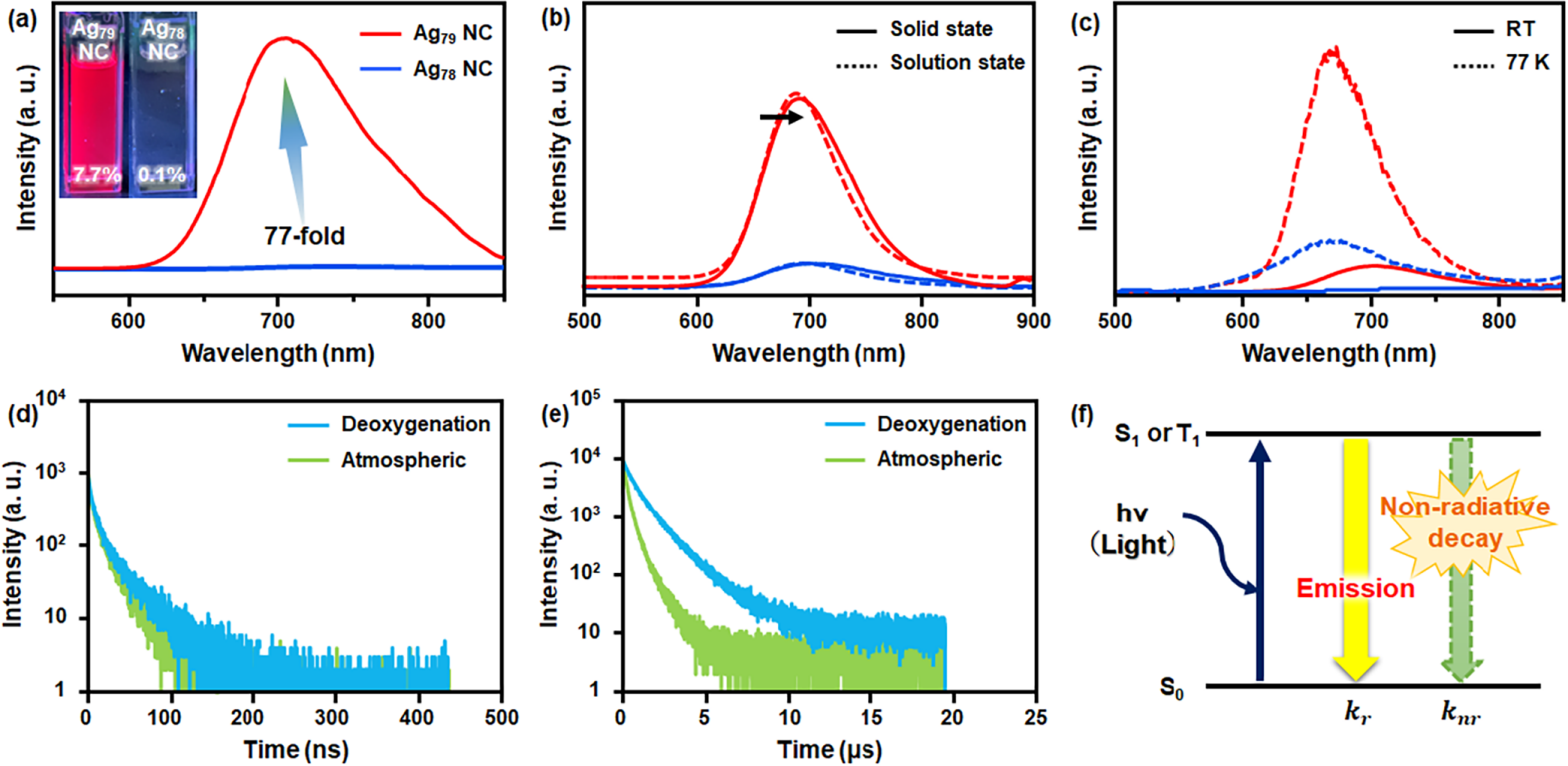
(a) Room-temperature PL emission for both Ag_78_ and Ag_79_ NC in the solution medium. The inset shows the excitation of the solutions from both NCs under UV-light irradiation. (b) Change in the difference in PL emission of both NCs between the solid state and the solution state. (c) Comparison in the PL emission intensities of both NCs at RT and 77 K. (d) PL decay curve of Ag_78_ NC under atmospheric and deoxygenated conditions. (e) PL decay curve of Ag_79_ NC under atmospheric and deoxygenated conditions. (f) Schematic representation of PL emission enhancement Ag_79_ NC through the synergistic effect.

The emission lifetimes of these NCs were measured under both air and deoxygenated conditions using time-correlated single-photon counting (TCSPC) (Figure 5d,e). In both cases, the NCs exhibited triexponential decay profiles, although a substantial difference in average emission lifetimes (τ_av_) was noted (Table S16). Under deoxygenated condition, it displayed a considerably longer τ_av_ of 1.144 μs, whereas Ag_78_ NC exhibited a much shorter τ_av_ of 22.45 ns. Notably, the presence of air had a detrimental effect, reducing the τ_av_ of both NCs. To further explore the effect of oxygen on emission lifetime, we calculated both the oxygen quenching coefficient and the Stern–Volmer coefficient (Table S17).^51^ The findings from these calculations suggest that the Ag_79_ NC is more prone to quenching caused by molecular oxygen than the Ag_78_ NC. This indicates that the emission process of Ag_79_ NC likely involves the participation of an excited triplet state. Moreover, recent research has highlighted that the emission characteristics of high-nuclear metal NCs, involve a process of photoexcitation that leads to the formation of a dark excited singlet state, which subsequently undergoes intersystem crossing (ISC) to a bright excited triplet state, resulting in phosphorescence.^52–55^ So, the longer emission lifetime of Ag_79_ NC is associated with the phosphorescence behavior.

To assess the different emission intensities of these NCs, the radiative (*k*_r_) and nonradiative (*k*_nr_) rate constants of individual NCs were estimated using their PL QY and emission lifetime values (Table S14).^21,56^ As we stated earlier, an increase in PL QY can be achieved either by enhancing the radiative decay or by suppressing the nonradiative decay channels. For Ag_79_ NC, a synergistic enhancement in PL QY is observed due to a 1.5-fold increase in *k*_r_ and a nearly two-order-of-magnitude reduction in *k*_nr_, in comparison to Ag_78_ NC. The enhancement in the *k*_r_ is attributed to the loss of *C*_3_ symmetry when transitioning from Ag_78_ NC to Ag_79_ NC, induced by the incorporation of an additional Ag atom on the outermost surface.^32^ This structural change leads to an increased oscillator strength (*f* = 0.005) (Table S11) for Ag_79_ NC compared to Ag_78_ NC (*f* ≈ 0) (Table S10), which is directly proportional to the *k*_r_, reinforcing the elevation of the radiative decay pathway in Ag_79_ NC compared to Ag_78_ NC.^57,58^ Furthermore, the increased oscillator strength also promotes stronger spin-forbidden ISC, which enhances the emission QY in conjunction with the increased *k*_r_ and τ_av_ for Ag_79_ NC compared to Ag_78_ NC. On the other hand, the suppression of the nonradiative decay in Ag_79_ NC is likely linked to the reduction in the vibrational relaxation process. This effect is attributed to enhanced surface rigidity on Ag–S/Ag–Ag skeleton, imposed by the presence of a tridentate ligand system and the additional Ag atom, along with additional thiolate ligand on the shell. So, this structural reinforcement minimizes the probability of nonradiative energy dissipation in the excited states, further contributing to the higher PL QY observed for Ag_79_ NC.

Although theoretical calculations indicate only a minimal difference in the absorption spectra of Ag_78_ and Ag_79_ NCs, notable variations in electron density distributions are observed in their excited states (Figure 6a). Specifically, the electronic transitions in Ag_78_ NC exhibit a greater degree of delocalization across the entire NC. This increased delocalization leads to a slight blue shift in the emission wavelength and a reduction in emission QY in Ag_78_ NC. In contrast, the additional Ag­(I) and ligands in Ag_79_ NCs induce structural distortions that modulate electron density, resulting in more localized transitions. This localization effect enhances the radiative recombination process, thereby contributing to a higher emission QY and a comparatively shorter emission wavelength. Furthermore, experimental results suggest that the emission mechanism in these NCs involves triplet excited states, indicating a possible role of ISC in their photophysical behavior. To gain deeper insight into the emission energy of these NCs, the geometry of the *T*_1_ states was optimized theoretically. The calculated phosphorescence energies for the NCs were relatively low, particularly for Ag_79_, with values of 1135 and 867 nm for Ag_78_ NC, respectively (Table S18). However, these results do not allow for the precise identification of the emitting states through calculations. Furthermore, the geometric changes during the T_1_-S_0_ transition for Ag_79_ NC were investigated through theoretical calculations, revealing an RMSD of 0.30 Å between the equilibrium geometries in the S_0_ and T_1_ states (Figures 6b and S30). This finding provides clear evidence of a significant structural transformation between the two electronic states. Additionally, the experimentally observed large Stokes shift (134 nm) for Ag_79_ NC further supports the occurrence of a distinct structural change in the excited state. To further investigate the nature of these structural variations, separate RMSD calculations were conducted for both the metallic Ag core and the metal–ligand-protected outer framework. The RMSD for the Ag core was calculated to be 0.23 Å, indicating a moderate structural shift, while the remaining parts of the structure, including the ligand shell, exhibited a slightly higher RMSD of 0.31 Å (Figure 6b). These findings suggest that the geometric rearrangements are not localized to a specific region but are rather distributed across the entire NC, affecting both the core and the outer framework. This comprehensive structural transformation at the excited state, coupled with the synergetic effect of enhanced *k*_r_ and reduced *k*_nr_ collectively drives the overall changes in the emission property of Ag_79_ NC compared to Ag_78_ NC, as depicted in Figure 5f. Based on their structural stability and corresponding photophysical behavior, we anticipate that these NCs hold significant potential for practical applications. To further assess this potential, we compared their PL QY with relevant literature reports on high-nuclearity Ag NCs (Table S19), as well as with other high-nuclearity NCs, alloy NCs, and related Ag_2_S nanoparticles. While Ag_2_S nanoparticles generally exhibit very low PL QY and require additional surface modifications to enhance emission, our approach not only leverages atomically precise structures but also achieves a strong correlation with high PL QY.

**Figure 6:**
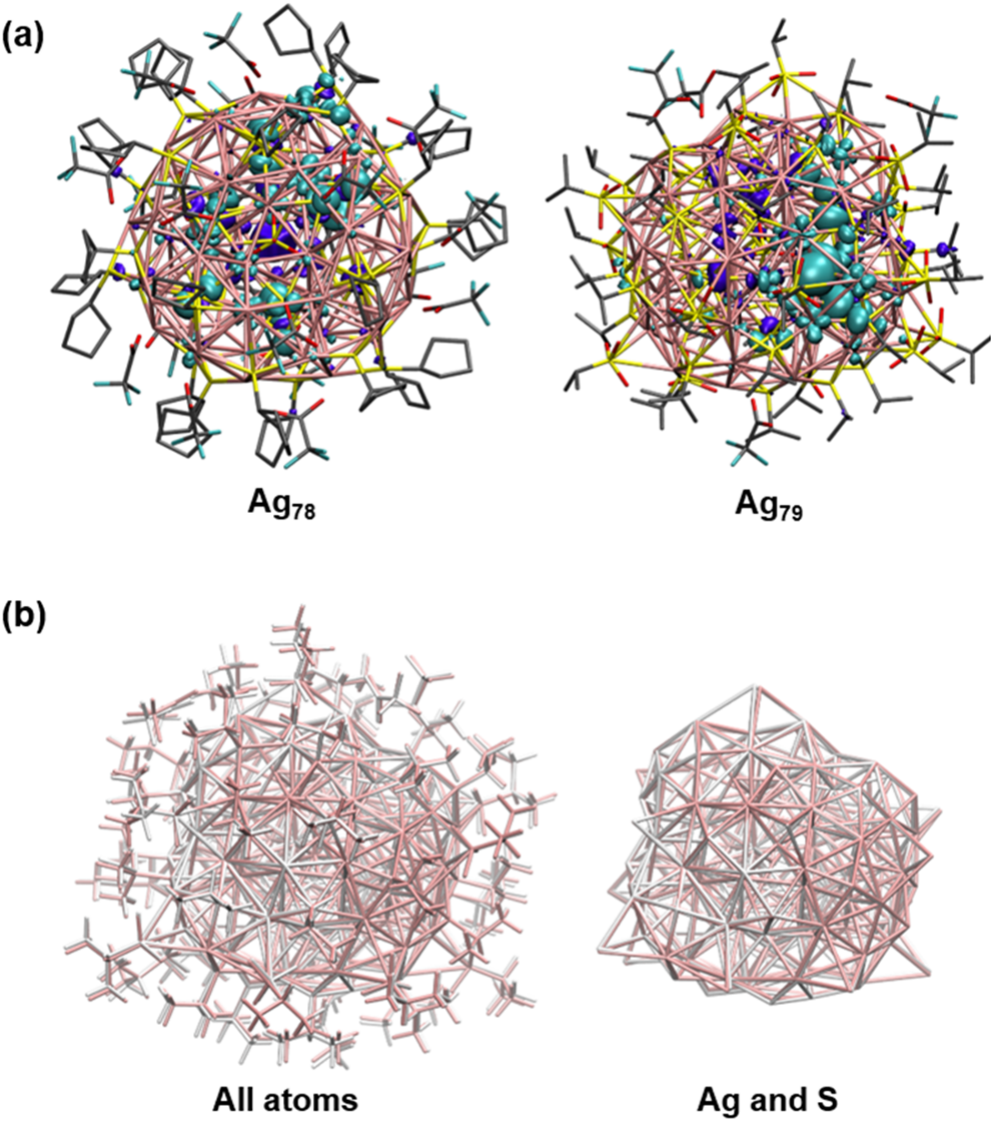
(a) Electron density difference between S_0_ and S_1_ for Ag_78_ and Ag_79_; increment (blue) and decrement (cyan). The iso-surface value is |0.0005| a.u. Those of other states are in Figures S25 and S26. (b) Comparison of the structures of Ag_79_ NC in the singlet (white) and triplet (pink) states. The superpositions of the structures were calculated based on the Kabsch algorithm.

## Conclusions

In conclusion, two high-nuclearity Ag nanoclusters, Ag_78_ and Ag_79_, were successfully synthesized and their geometric structures were determined by SCXRD. Detailed analysis revealed that both NCs have very similar geometric structures, with the only difference being the incorporation of a single Ag atom within the ligand shell of Ag_78_. The incorporation of this additional Ag atom was achieved through modifications of the surface-protecting ligand system, which influenced its coordination environment within the ligand shell of the cluster. This strategic alteration modified argentophilic interactions and generated a localized void, allowing the accommodation of the extra Ag atom along with its ligand protection, without disturbing the overall core–shell geometry. However, the introduction of the additional Ag atom induced a reduction in the overall symmetry of the NC while simultaneously increasing the rigidity of its surface framework. These structural modifications had a synergistic effect on the PL properties by influencing both radiative and nonradiative decay pathways. The enhanced rigidity suppressed nonradiative decay processes by restricting atomic fluctuations, while a reduction in symmetry facilitated radiative transitions, thereby leading to improved emission characteristics. So, this study provides a fundamental understanding of how precise atomic-level modifications in the ligand shell can simultaneously regulate both key mechanistic factors governing PL in Ag NCs. By demonstrating a viable approach for fine-tuning the structural and electronic architectures of metal NCs, these findings offer valuable insights into the rational design of highly emissive Ag NCs for advanced optoelectronic and sensing applications.

## Supplementary Material



## Notes

Experimental section; crystal parameter tables; theoretical calculation tables; ESI-MS table; emission lifetime table; oxygen quenching coefficient and the Stern–Volmer coefficient of both NCs; radiative and nonradiative parameters; optical microscope image of crystals; coordination of the templating anion with the Ag atoms; position of the [S]^2–^ ligands, five-layer distribution of [S]^2–^ ligands; position of the inward Ag atoms in the outermost shell of both NCs; arrangement of outermost Ag atoms in outermost shell of both NCs; layers of the outermost cationic shells for both NCs; ligand coordination in Ag_78_ NC; ligand coordination in Ag_79_ NC; different Ag–Ag bond length at different ligand coordination site; inter cluster interaction for both NCs; theoretical structural architecture comparison; ESI-MS of both NCs; FT-IR spectra of both NCs, XPS of both NCs; time-dependent UV in solution; MOs of both NCs; electron density differences between ground and excited states of both NCs; molar absorption coefficient of both NCs in solution medium; temperature-dependent PL of both NCs; and comparison of the structures of Ag_79_ NC in the singlet and triplet states by theoretical calculation (PDF)
