# Supplementary material for: Triggering Photoluminescence in High-Nuclear Silver Nanoclusters via Extra Silver Atom Incorporation
Source: J Am Chem Soc. 2025 Sep 30;147(41):37231–41. doi: 10.1021/jacs.5c10289 (PMC12532279; doi:10.1021/jacs.5c10289)
Supplement: Supplementary file 1 [file ja5c10289_si_001.pdf]

Supporting Information

Triggering Photoluminescence in High-Nuclear Silver Nanoclusters via Extra Silver Atom Incorporation

Aoi Akiyama,[a]† Sakiat Hossain,[b]† Sourav Biswas,*[c] Takafumi Shiraogawa,[d] Pei Zhao,[d] Mana Nakamoto,[a] Daiji Ogata,[a] Tokuhisa Kawawaki,[c] Yoshiki Niihori,[b] Junpei Yuasa,[a] Masahiro Ehara,*[d] Yuichi Negishi*[a][b][c]

[a] Department of Applied Chemistry, Tokyo University of Science,1-3 Kagurazaka, Shinjuku-ku, Tokyo 162-8601, Japan.

[b] Research Institute for Science & Technology, Tokyo University of Science, Tokyo 162-8601, Japan.

[c] Institute of Multidisciplinary Research for Advanced Materials, Tohoku University, Katahira, Sendai 980-8577, Japan.

[d] Institute for Molecular Science, 38, NishigoNaka, Myodaiji, Okazaki-shi, Aichi 444-8601, Japan.

† A.A. and S.H contributed equally to this paper.

*Corresponding Authors

S.B.: sourav.biswas210@gmail.com

M.E.: ehara@ims.ac.jp

Y.N.: yuichi.negishi.a8@tohoku.ac.jp

Table of Contents

**Name**	**Description**	**Page No.**
	Experimental section	S4-S8
Table S1	Crystal data and structure refinement parameters of Ag_{78} NC	S9
Table S2	Crystal data and structure refinement parameters of Ag_{79} NC	S10
Table S3	WBO bond length for Ag_{78} NC	S11
Table S4	WBO bond length for Ag_{79} NC	S12
Table S5	NPA charges for Ag_{78} NC	S13-S14
Table S6	NPA charges for Ag_{79} NC	S14-S15
Table S7	EDA-NOCV results for Ag_{78} NC	S16
Table S8	EDA-NOCV results for Ag_{79} NC	S16
Table S9	ESI-MS peaks for both NCs	S17
Table S10	Selected excited states of Ag_{79} NC	S17
Table S11	Selected excited states of Ag_{78} NC	S18
Table S12	Contributions of the atomic species and Ag and S atoms to MOs of Ag_{78} NC	S18-S19
Table S13	Contributions of the atomic species and Ag and S atoms to MOs of Ag_{79} NC	S19
Table S14	Radiative and noon-radiative parameters	S20
Table S15	Temperature dependence emission parameters	S20
Table S16	Emission lifetimes for both NCs	S20
Table S17	Oxygen quenching coefficient and the Stern-Volmer coefficient of both NCs	S20
Table S18	Total energy at the T_1-state geometry	S20
Table S19	Comparison of PL emission QY with the reported literature	S21
Figure S1	Optical microscope image of both crystals and reaction scheme	S22
Figure S2	Position of the $[S]^{2-}$ ligands	S22
Figure S3	Five-layer distribution of $[S]^{2-}$ ligands	S23
Figure S4	Position of the inward Ag atoms in the outer most shell of both NCs	S23
Figure S5	Arrangement of outer most Ag atoms in outermost shell of both NCs	S24
Figure S6	Layers of the outermost cationic shells for both NCs	S24
Figure S7	C_3 symmetry axis of Ag_{78} NC	S25

Figure S8	Ligand coordination in Ag_{78} NC	S25
Figure S9	Ligand coordination in Ag_{79} NC	S26
Figure S10	Different Ag-Ag bond length at different ligand coordination site	S26
Figure S11	Inter cluster interaction for both NCs	S27
Figure S12	Theoretical structural architecture comparison	S27
Figure S13	ESI-MS of Ag_{78} NC	S28
Figure S14	ESI-MS of Ag_{79} NC	S28
Figure S15	ESI-MS of Ag_{79} NC with different concentration	S28
Figure S16	Shifting of the equilibrium in ESI-MS of Ag_{79} NC with different concentrations	S29
Figure S17	FT-IR spectra of both NCs	S29
Figure S18	XPS survey spectrum for both NCs	S29
Figure S19	Deconvoluted XPS data of Ag_{78} NC	S30
Figure S20	Deconvoluted XPS data of Ag_{79} NC	S30
Figure S21	Time dependent UV in solution	S31
Figure S22	Photostability of Ag_{78} and Ag_{79} NCs	S31
Figure S23	DFT functional dependence of the absorption spectrum of Ag_{79} NC	S32
Figure S24	MOs relevant to the absorption spectrum of Ag_{78} NC	S33
Figure S25	MOs relevant to the absorption spectrum of Ag_{79} NC	S34
Figure S26	Electron density differences between ground and excited states of Ag_{79} NC	S35
Figure S27	Electron density differences between ground and excited states of Ag_{78} NC	S36
Figure S28	Molar absorption coefficient of both NCs in solution medium	S37
Figure S29	Temperature dependent PL of both NCs	S37
Figure S30	Comparison of the structures of Ag_{79} NC in the singlet and triplet states by theoretical calculation	S37
	References	S38-S39

Experimental Section:

Reagents

All chemicals were obtained commercially and were used without further purification. Silver(I) Trifluoroacetate (CF_3CO_2Ag), Copper(II) Nitrate Trihydrate (($CuNO_3)_2$ · $3H_2O$), Triethylamine were from FUJIFILM Wako Pure Chemical Co. Cyclopentanethiol (CpSH), 2-Propanethiol (^{i}PrSH) were from Tokyo Chemical Industry Co. Silver nitrate ($AgNO_3$), Diethyl ether were from Kanto Chemical Co., Inc. Oxazine 1 perchlorate were from Med Chem Express.

Synthesis of Cyclopentane silver thiolate complex $[CpSAg]_n$

The solution of $AgNO_3$ (3.0 mmol, 510 mg) in 7.5 mL acetonitrile was mixed with 10 mL ethanol containing CpSH (3.0 mmol, 0.32 mL) and 0.5 mL Et_3N under stirring for 3 hours in the dark at room temperature, then the yellow powder of $[CpSAg]_n$ was isolated by filtration and washed with 10 mL ethanol and 20 mL ether, then dried in the ambient environment.[S1] $[CpSAg]_n$ was obtained in 95% yield.

Synthesis of Ag_{78} NC

Ag_{78} NC was synthesized by following a previously reported procedure with modifications. Typically, the reaction was carried out by mixing the previously synthesized $[CpSAg]_n$ (21 mg) complex and CF_3COOAg (24.3 mg, 0.11 mmol) in MeOH/CH_2Cl_2 (5 mL, v/v 2 : 1) in the presence of Na_2SO_4 (5.7 mg, 0.04 mmol) which gave a yellow solution as represented in Scheme S1.[S1] Here it is important to note that the presence of soluble CF_3COOAg is crucial for the depolymerization of $(CpSAg)_n$ in a MeOH/CH_2Cl_2 mixture. The insoluble reactants and byproducts were filtered out, and the resulting solution was collected and stored in the dark in a capped crystallization vial. Dark-brown plate-like crystals formed over the course of two weeks. These were collected and dried at room temperature. It is also worth mentioning that the overall change in solvent volume in the capped vial was negligible during the crystallization period. The Ag_{78} nanocluster was obtained with a yield of approximately 15% (based on Ag metal).

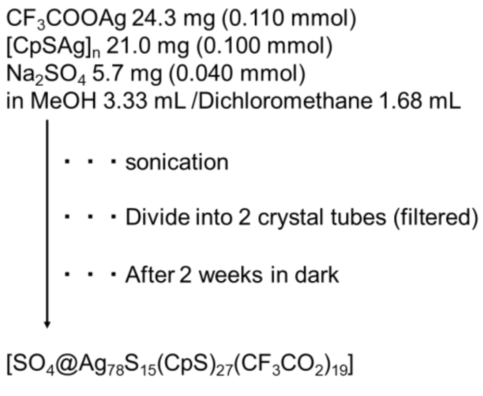

Scheme S1. Schematic representation of the reaction flow chart for Ag_{78} NC synthesis.

Synthesis of Ag_{79} NC

Similar synthetic approach as synthesis of Ag_{78} using varying ratios of $(RSAg)_n$ (where R= -^{i}Pr, -Cp) , CF_3COOAg and Na_2SO_4 did not lead to neither Ag_{79} nor any new characterizable NCs. Subsequently, iso-propyl mercaptan (0.13 mmol) was directly treated with CF_3COOAg in a solvent mixture of 2 mL acetone and 2 mL acetonitrile. However, this modified condition also did not yield the desired result until $Cu(NO_3)_2{\cdot}3H_2O$ was introduced into the reaction mixture. To a reaction mixture of CF_3COOAg (0.220 mmol) and $Cu(NO_3)_2{\cdot}\ 3H_2O$ (10.6 mg, 0.044 mmol) in 10.5 ml acetone and 10.5 ml acetonitrile, iPr-SH was added and stirred for about 10 S. Color of the solution changes from initial blue to faint blue, indicating reaction of ^{i}PrSH with Cu(II) ions and in-situ formation of $^iPrSO_3^-$ ligand and SO_4^{2-} templating anion. Then the reaction mixture was divided in crystal vials which were loosely capped and kept under fluorescent light in air at room temperature. Color of the solution gradually changes back from faint blue to deep blue as days go by, indicating aerobic oxidation of colorless Cu(I) to blue color Cu(II). After one month, the brown-black crystals were formed on the glass surface. The Ag_{79} NC was obtained with a yield of approximately 5% (based on Ag metal). Here it is important to mention that the addition of Na_2SO_4 in various ratios, as used in the previous synthetic protocol, did not result in the formation of Ag_{79} or any other NCs.

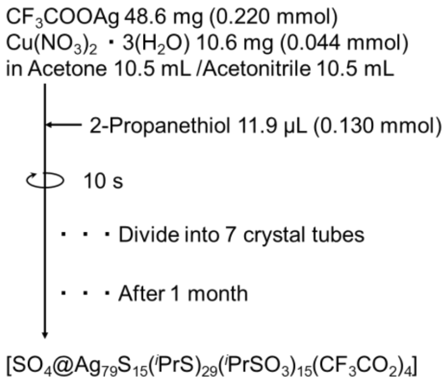

Scheme S2. Schematic representation of the reaction flow chart for Ag_{79} NC synthesis.

Instruments

Optical absorption spectroscopy

The optical absorption spectrum of the dichloromethane solution of Ag_{78} or Ag_{79} was obtained at room temperature with a V-630 spectrometer (JASCO, Tokyo, Japan). Multiple crystals were dissolved in dichloromethane for the measurement.

The diffuse reflection spectroscopy

The diffuse reflection spectroscopy was acquired at ambient temperature with a V-670 and V-770spectrometer (JASCO, Tokyo, Japan) by mixing the synthesized material with $BaSO_4$. The

wavelength-dependent optical data (I(w)) were converted to energy-dependent data (II) by the following equation that conserved the integrated spectral areas: II = I(w)/|∂E/∂w| ∝ I(w) × w2.

XPS

X-ray photoelectron spectroscopy (XPS) was performed using JEOL's JPS-9010MC. The sample was placed on an indium plate, irradiated with Mg-Kα (1253.6 eV) radiation under reduced pressure of ~2 × 10^{-8} Torr, and the photoelectron spectra were measured. After measurement, the spectra were corrected using reference values for In $3d_{5/2}$ (443.8 eV).[S2]

Fourier Transform Infrared Absorption Spectroscopy

Fourier transform infrared (FT-IR) absorption spectra were recorded using a JASCO FT/IR-4600 spectrometer. Sample was dissolved in dichloromethane and deposited onto a diamond prism, followed by solvent evaporation. The spectrum of the prepared sample was then measured in the range of 400 to 4000 cm^{-1}.

Electrospray Ionization Mass Spectrometry

For the infusion analysis by electrospray ionization mass spectrometry (ESI-MS), we used MicroToF, Bruker and otofControl system. Sample was dissolved in toluene containing acetone and electrospray ionization mass spectrometry was attempted in positive ion mode. The spray voltage was set to 6000 V, the desolvation temperature to 200 °C, and the flow rate to 200 μL/h.

Photoluminescence Spectroscopy and Their Analysis

First, sample was dissolved in toluene and the solution was poured into quartz cuvette with optical length of 1.0 cm. Oxygen molecules in the solution were removed from the solution by bubbling argon containing saturated toluene vapor. The emission spectra of sample ($I_X(\lambda_{em})$) were measured under deoxygenated conditions by irradiation of 405 nm excitation light using JASCO's FP-6300 and the absorbance $A(\lambda_{ex})$ at the excitation wavelength was measured by JASCO's V-770. The emission spectra (F_{norm}) normalized by absorptance was obtained using the following equation:

$$\boldsymbol{F}_{\mathbf{norm}}(\boldsymbol{\lambda}_{\mathbf{em}}) = \frac{\boldsymbol{I}_{\mathbf{X}}(\boldsymbol{\lambda}_{\mathbf{em}})}{\mathbf{1} - \mathbf{10}^{-\boldsymbol{A}(\boldsymbol{\lambda}_{\mathbf{ex}})}}. \qquad \textbf{(Equation S1)}$$

The photoluminescence quantum yield (PLQY, Φ_{PL}) was determined by the relative method[S3] using Oxazine 1 (Φ_{std} = 0.141 in deaerated ethanol)[S4] as a standard sample. The obtained values were substituted into the following formula to evaluate the relative spectral quantum yield of the unknown sample X.

$$\Phi_{\mathrm{PL,X}} = \frac{1 - 10^{-A_{\mathrm{std}}(\lambda_{\mathrm{ex}})}}{1 - 10^{-A_{\mathrm{X}}(\lambda_{\mathrm{ex}})}} \cdot \frac{\int I_{\mathrm{X}}(\lambda_{\mathrm{em}})\mathrm{d}\lambda_{\mathrm{em}}}{\int I_{\mathrm{std}}(\lambda_{\mathrm{em}})\mathrm{d}\lambda_{\mathrm{em}}} \cdot \left(\frac{n_{\mathrm{X}}}{n_{\mathrm{std}}}\right)^2 \cdot \Phi_{\mathrm{std}}. \qquad \textbf{(Equation S2)}$$

, where λ_{ex} and λ_{em} are the excitation and emission wavelengths, respectively, n is the refractive index of the solvent ($n_{dichloromethane}$ = 1.424, $n_{ethanol}$ = 1.361). The integration of PL spectrum represents the area of PL spectrum.

Photoluminescence Lifetime

PL lifetimes were measured with a HORIBA Scientific time-correlated single-photon counting system (FluoroCube) with a pulsed LED source (452 nm) as excitation source. Sample was dissolved in toluene and the solution was degassed by bubbling with argon containing saturated toluene vapor. The PL decay curve $I_{PL}(t)$ was fitted by the least-squares method using the following multiple exponential function:

$$I_{\mathrm{PL}}(t) = \sum_{i=1}^{3} A_i \exp\left(-\frac{t}{\tau_i}\right) + B. \qquad \textbf{(Equation S3)}$$

, where A is the amplitude (pre-exponential factor), B is the background, and τ is the emission lifetime. The fractional amplitude α and fractional population f were calculated as follows:

$$\alpha_i = \frac{A_i}{\sum_{j=1}^{3} A_j}, \qquad f_i = \frac{\alpha_i \tau_i}{\sum_{j=1}^{3} \alpha_j \tau_j}. \qquad \textbf{(Equation S4)}$$

The lifetime was evaluated as the average PL lifetime based on the number of emitted photons using the following formula:

$$\tau_{\mathrm{PL}} = \sum_{i=1}^{3} f_i \tau_j \qquad \textbf{(Equation S5)}$$

Radiative and Non-radiative Rate Constant

The radiative and non-radiative rate constant (k_r and k_{nr}, respectively) were estimated by the following equations.

$$k_{\mathrm{r}} = \frac{\Phi_{\mathrm{PL}}}{\tau_{\mathrm{PL}}}, \qquad k_{\mathrm{nr}} = \frac{1-\Phi_{\mathrm{PL}}}{\tau_{\mathrm{PL}}}. \qquad \textbf{(Equation S6)}$$

The radiative lifetime (τ_r) is a reciprocal of k_r as follows.

$$\tau_{\mathrm{r}} = \frac{1}{k_{\mathrm{r}}}. \qquad \textbf{(Equation S7)}$$

X-ray Crystallography details

A single crystal was immersed in the cryoprotectant Parabar 10312 (Hampton Research, 34 Journey, Aliso Viejo, CA 92656-3317 USA) and kept at 90 K during diffraction data collection. A Bruker D8

QUEST diffractometer was used to collect the diffraction data for the single crystal using monochromated Mo Kα radiation (λ = 0.71073 Å). Although many crystals from different batches were checked for the diffraction experiment, all of them lacked higher angle data. However, the collected diffraction data was good enough to obtain a core structure containing Ag(I), S^{2-} and a partial Ag(I)-ligand shell, which was solved by SHELXT[S5] using the intrinsic phasing method in Apex4 Bruker Software Suite.[S6] Later, during refinement the full crystal structure was completed using the full-matrix least squares method against F2 by SHELXL-2018/3 in Olex2 GUI[S7]. Although a few minor check cif alerts are there, we have provided comprehensive responses embedded within each CIF file.

Computational details

The electronic structures of the real Ag clusters, Ag_{78} and Ag_{79} NCs were calculated by the DFT and TDDFT calculations without simplifying the structures. Based on the systematic examinations of the functionals in the previous work of anion-templated Ag clusters[S8], MN15 functional[S9] was adopted for the present DFT/TDDFT calculations. The relativistic effective core potential (RECP) LANL2DZ[S10] was adopted for Ag atoms and the basis sets of 6-31G(d) was selected for H, C, O, F, and S atoms[S11] for the ground-state geometry optimizations. For simulating the absorption spectra, TDDFT calculations were carried out and 50 excited states were solved to cover the energy range up to ~300 nm (for both NCs). For the TDDFT calculations, the basis sets of 3-21G* (S) and 3-21G (H, C, O, F) were adopted because of the computational costs of these large clusters. All the DFT and TDDFT calculations were conducted using the Gaussian 16 suite of programs version C.2.[S12]

The NPA charges and Wiberg bond orders were calculated using the B3LYP functional combined with the basis sets of 3-21G*/6-31G* (S, C, O, F, and H) and LANL2DZ (Ag) using NBO 3.1.[S13-S14] The information on the singly excited states was obtained with cclib[S15]. Mulliken partitioning of MOs into atomic contributions and calculations of electron density differences were performed using Multiwfn[S16]. RMSDs for geometries were calculated with the Kabsch algorithm[S17] using a partially modified rmsd code[S18]. All structures, MOs, and electron densities were visualized with VMD.8.[S19]

Table S1. Crystal data and structure refinement parameters of Ag_{78} NC.

Identification code	Ag78
Empirical formula	$C_{202.8}H_{243}Ag_{78}F_{27}O_{41.92}S_{41.98}$
CCDC number	2427981
Formula weight	13624.02
Temperature/K	90.15
Crystal system	trigonal
Space group	P-3
a/Å	22.6442(9)
b/Å	22.6442(9)
c/Å	37.540(2)
α/°	90
β/°	90
γ/°	120
Volume/$Å^3$	16670.2(16)
Z	2
ρ_{calc}/g cm^{-3}	2.714
μ/mm^{-1}	4.785
F(000)	12752.0
Crystal size/mm^3	0.7 × 0.6 × 0.4
Radiation	MoKα (λ = 0.71073)
2Θ range for data collection/°	3.758 to 41.74
Index ranges	$-22 \le h \le 22$, $-22 \le k \le 22$, $-37 \le l \le 37$
Reflections collected	137876
Independent reflections	11727 [R_{int} = 0.0681, R_{sigma} = 0.0291]
Data/restraints/parameters	11727/3031/1125
Goodness-of-fit on F^2	2.112
Final R indexes [I>=2σ (I)]	R_1 = 0.1334, wR_2 = 0.4109
Final R indexes [all data]	R_1 = 0.1688, wR_2 = 0.4665
Largest diff. peak/hole / e $Å^{-3}$	7.77/-3.44

Table S2. Crystal data and structure refinement parameters of Ag_{79} NC.

Identification code	Ag79
Empirical formula	$Ag_{79}C_{132}F_{12}O_{57}S_{59}H_{243}$
CCDC number	2427980
Formula weight	13383.52
Temperature/K	90(2)
Crystal system	monoclinic
Space group	C2/c
a/Å	78.6522(18)
b/Å	22.1968(5)
c/Å	38.8719(9)
α/°	90
β/°	114.7320(10)
γ/°	90
Volume/Å^3	61639(2)
Z	8
ρ_{calc}/g cm^{-3}	2.884
μ/mm^{-1}	5.342
F(000)	50048.0
Crystal size/mm^3	0.400 × 0.300 × 0.250
Radiation	MoKα (λ = 0.71073)
2Θ range for data collection/°	3.744 to 50.812
Index ranges	$-94 \le h \le 94$, $-26 \le k \le 21$, $-46 \le l \le 46$
Reflections collected	314881
Independent reflections	56602 [R_{int} = 0.0488, R_{sigma} = 0.0353]
Data/restraints/parameters	56602/1102/2943
Goodness-of-fit on F^2	1.058
Final R indexes [I>=2σ (I)]	R_1 = 0.0928, wR_2 = 0.2369
Final R indexes [all data]	R_1 = 0.1263, wR_2 = 0.2752
Largest diff. peak/hole / e Å^{-3}	7.75/-4.28

Table S3. Bond length (d, Å) and Wiberg bond order (WBO) of S–O (central SO_4) and O···Ag (between the O atoms of SO_4 and the closest Ag atoms) in Ag_{78} NC.

S–O/O···Ag	*d*	WBO	
		3-21G*	6-31G*
S1–O2	1.494	1.061	1.055
S1–O3	1.512	0.995	0.985
S1–O4	1.512	0.995	0.985
S1–O5	1.512	0.995	0.985
O2···Ag15	2.755	0.039	0.032
O2···Ag16	2.755	0.039	0.032
O2···Ag69	2.755	0.039	0.032
O3···Ag74	2.703	0.027	0.020
O3···Ag81	2.653	0.045	0.037
O3···Ag129	2.430	0.031	0.022
O3···Ag135	2.922	0.024	0.018
O4···Ag6	2.703	0.027	0.020
O4···Ag11	2.653	0.045	0.036
O4···Ag51	2.430	0.031	0.022
O4···Ag52	2.922	0.024	0.018
O5···Ag12	2.430	0.031	0.022
O5···Ag46	2.703	0.027	0.020
O5···Ag55	2.922	0.024	0.018
O5···Ag59	2.653	0.045	0.037

Table S4. Bond length (d, Å) and Wiberg bond order (WBO) of S–O (central SO_4) and O···Ag (between the O atoms of SO_4 and the closest Ag atoms) in Ag_{79} NC.

S–O/O···Ag	*d*	WBO	
		3-21G*	6-31G*
S1–O2	1.513	1.006	0.999
S1–O3	1.509	1.021	1.014
S1–O4	1.505	1.053	1.047
S1–O5	1.520	0.984	0.974
O2···Ag6	2.800	0.065	0.048
O2···Ag17	2.665	0.031	0.025
O2···Ag21	2.595	0.028	0.021
O2···Ag23	2.796	0.029	0.022
O3···Ag13	2.592	0.033	0.027
O3···Ag31	2.661	0.029	0.023
O3···Ag33	2.895	0.030	0.022
O3···Ag41	2.951	0.032	0.024
O4···Ag37	2.680	0.030	0.021
O4···Ag43	2.829	0.030	0.024
O4···Ag47	2.998	0.032	0.027
O4···Ag49	2.932	0.028	0.022
O4···Ag74	2.994	0.031	0.022
O5···Ag9	2.689	0.038	0.030
O5···Ag11	2.885	0.035	0.029
O5···Ag19	2.517	0.037	0.028
O5···Ag21	2.873	0.029	0.021
O5···Ag37	2.811	0.032	0.024

Table S5. The NPA charges of Ag_{78} NC.

Atom No.	Atom type	NPA charge		Atom No.	Atom type	NPA charge	
		3-21G*	6-31G*			3-21G*	6-31G*
1	S	2.577	2.745	71	Ag	0.484	0.551
2	O	–0.866	–0.967	72	Ag	0.510	0.583
3	O	–0.900	−1.009	73	Ag	0.490	0.562
4	O	–0.900	−1.009	74	Ag	0.397	0.451
5	O	–0.900	−1.009	75	Ag	0.484	0.551
6	Ag	0.397	0.451	76	Ag	0.510	0.583
10	Ag	0.334	0.401	77	Ag	0.490	0.562
11	Ag	0.350	0.408	79	Ag	0.591	0.671
12	Ag	0.415	0.467	81	Ag	0.350	0.408
13	Ag	0.477	0.558	82	Ag	0.477	0.558
14	Ag	0.476	0.535	83	Ag	0.476	0.535
15	Ag	0.322	0.386	97	Ag	0.505	0.587
16	Ag	0.322	0.386	129	Ag	0.415	0.467
17	Ag	0.484	0.551	130	Ag	0.477	0.558
18	Ag	0.510	0.583	131	Ag	0.476	0.535
19	Ag	0.490	0.562	132	Ag	0.645	0.693
20	Ag	0.334	0.401	135	Ag	0.384	0.442
21	Ag	–0.016	0.062	136	Ag	0.409	0.473
22	Ag	0.485	0.563	137	Ag	0.485	0.548
23	Ag	0.463	0.528	138	Ag	0.376	0.444
24	Ag	0.590	0.655	139	Ag	0.564	0.628
46	Ag	0.397	0.451	140	Ag	0.505	0.587
47	Ag	–0.016	0.062	142	Ag	0.550	0.633
48	Ag	0.486	0.563	144	Ag	0.409	0.473
49	Ag	0.463	0.528	145	Ag	0.485	0.548
50	Ag	0.590	0.655	146	Ag	0.376	0.444
51	Ag	0.415	0.467	148	Ag	0.550	0.633
52	Ag	0.384	0.442	150	Ag	0.564	0.628
53	Ag	0.413	0.467	162	Ag	0.016	0.062

54	Ag	0.263	0.319	163	Ag	0.486	0.563
55	Ag	0.384	0.442	164	Ag	0.463	0.528
56	Ag	0.409	0.473	165	Ag	0.590	0.655
57	Ag	0.485	0.548	166	Ag	0.591	0.671
58	Ag	0.376	0.444	175	Ag	0.413	0.467
59	Ag	0.350	0.408	176	Ag	0.263	0.319
60	Ag	0.413	0.467	177	Ag	0.645	0.693
61	Ag	0.263	0.319	197	Ag	0.505	0.587
62	Ag	0.452	0.516	222	Ag	0.452	0.516
64	Ag	0.591	0.671	227	Ag	0.550	0.633
66	Ag	0.645	0.693	229	Ag	0.452	0.516
69	Ag	0.322	0.386	231	Ag	0.564	0.628
70	Ag	0.334	0.401				

Table S6. The NPA charges of Ag_{79} NC.

Atom No.	Atom type	NPA charge		Atom No.	Atom type	NPA charge	
		3-21G*	6-31G*			3-21G*	6-31G*
1	S	2.560	2.725	43	Ag	0.374	0.431
2	O	–0.892	–1.000	44	Ag	0.530	0.599
3	O	–0.880	–0.988	45	Ag	0.159	0.235
4	O	–0.864	–0.969	46	Ag	0.442	0.504
5	O	–0.900	–1.011	47	Ag	0.330	0.384
6	Ag	–0.059	0.031	48	Ag	0.472	0.543
7	Ag	0.436	0.502	49	Ag	0.402	0.458
8	Ag	0.625	0.681	50	Ag	0.546	0.604
9	Ag	0.302	0.365	51	Ag	0.322	0.398
10	Ag	0.562	0.619	52	Ag	0.441	0.505
11	Ag	0.341	0.392	53	Ag	0.493	0.578
12	Ag	0.601	0.669	54	Ag	0.493	0.557
13	Ag	0.387	0.439	55	Ag	0.529	0.591
14	Ag	0.489	0.551	56	Ag	0.522	0.601
15	Ag	0.169	0.257	57	Ag	0.438	0.515

16	Ag	0.433	0.499	58	Ag	0.489	0.549
17	Ag	0.389	0.444	59	Ag	0.467	0.533
18	Ag	0.502	0.580	60	Ag	0.486	0.547
19	Ag	0.372	0.430	61	Ag	0.513	0.575
20	Ag	0.521	0.596	62	Ag	0.535	0.604
21	Ag	0.401	0.459	63	Ag	0.536	0.590
22	Ag	0.485	0.555	64	Ag	0.490	0.549
23	Ag	0.398	0.449	65	Ag	0.450	0.518
24	Ag	0.492	0.566	66	Ag	0.415	0.486
25	Ag	0.416	0.473	67	Ag	0.599	0.657
26	Ag	0.371	0.439	68	Ag	0.452	0.523
27	Ag	0.503	0.566	69	Ag	0.390	0.460
28	Ag	0.485	0.551	70	Ag	0.511	0.598
29	Ag	0.346	0.402	71	Ag	0.440	0.506
30	Ag	0.581	0.669	72	Ag	0.542	0.610
31	Ag	0.396	0.449	73	Ag	0.434	0.499
32	Ag	0.461	0.533	74	Ag	0.348	0.413
33	Ag	0.397	0.453	75	Ag	0.469	0.537
34	Ag	0.483	0.548	76	Ag	0.465	0.531
35	Ag	0.323	0.387	77	Ag	0.542	0.617
36	Ag	0.479	0.540	78	Ag	0.630	0.697
37	Ag	0.414	0.472	79	Ag	0.447	0.513
38	Ag	0.461	0.522	80	Ag	0.507	0.576
39	Ag	0.457	0.527	81	Ag	0.550	0.601
40	Ag	0.491	0.553	82	Ag	0.518	0.585
41	Ag	0.375	0.438	83	Ag	0.410	0.480
42	Ag	0.374	0.457	84	Ag	0.520	0.585

Table S7. EDA-NOCV results (in kcal/mol) of the SO_4–Ag_{78} interaction in Ag_{78} NC. Values in parentheses are the percentage contribution to the total orbital interaction ΔE_{orb}.

	SO_4^{-2}@Ag_{78}^{+3} singlet-singlet	SO_4^{-1}@Ag_{78}^{+2} doublet-doublet
ΔE_{int}	–495.3	–288.8
ΔE_{Pauli}	304.6	314.1
ΔE_{disp}	–71.5	–71.5
ΔE_{elstat}	–505.5	–322.0
ΔE_{orb}	–222.8	–209.4
ΔE_{orb1}	–22.2(10.0%)	–81.1(38.7%)
ΔE_{orb2}	–22.1(9.9%)	–16.2(7.7%)
ΔE_{orb3}	–18.8(8.4%)	–14.0(6.7%)
ΔE_{orb4}	–18.1(8.1%)	–11.3(5.4%)

Table S8. EDA-NOCV results (in kcal/mol) of the SO_4–Ag_{79} interaction in Ag_{79} NC. Values in parentheses are the percentage contribution to the total orbital interaction ΔE_{orb}.

	SO_4^{-2}@Ag_{79}^{+2} singlet-singlet	SO_4^{-1}@Ag_{79}^{+1} doublet-doublet
ΔE_{int}	–387.4	–232.9
ΔE_{Pauli}	238.8	244.6
ΔE_{disp}	–70.4	–70.4
ΔE_{elstat}	–344.5	–224.2
ΔE_{orb}	–211.3	–182.9
ΔE_{orb1}	–23.6(11.2%)	–68.2(37.3%)
ΔE_{orb2}	–20.8(9.8%)	–15.4(8.4%)
ΔE_{orb3}	–17.2(8.1%)	–13.0(7.1%)
ΔE_{orb4}	–14.1(6.7%)	–10.4(5.7%)

Table S9. ESI-MS peaks (i) to (v).

Nanocluster	Peak number	m/z	Formula
Ag_{78}	(i)	6827.6	$[SO_4@Ag_{76}S_{15}(CpS)_{27}(CF_3CO_2)_{19}]^{2-}$
	(ii)	6931.0	$[SO_4@Ag_{77}S_{15}(CpS)_{28}(CF_3CO_2)_{19}]^{2-}$
Ag_{79}	(iii)	6479.8	$[SO_4@Ag_{78}S_{15}(^iPrS)_{30}(^iPrSO3)_{13}(CF_3CO_2)]^{2+}$
	(iv)	6522.2	$[SO_4@Ag_{78}S_{15}(^iPrS)_{28}(^iPrSO3)_{14}(CF_3CO_2)_2]^{2+}$
	(v)	6629.7	$[SO_4@Ag_{79}S_{15}(^iPrS)_{26}(^iPrSO_3)_8(CF_3CO_2)_{11}]^{2+}$

Table S10. Selected excited states of Ag_{78} NC with transition energy (ΔE), wavelength (*λ*), oscillator strength (*f*) and characters. (H, HOMO; L, LUMO).

State	Δ*E* (eV)	*λ* (nm)	*f*	Character	
1	2.37	523	0.001	H-1→L (0.74)	LMCT + LE
6	2.62	474	0.011	H-10→L (0.79)	LMCT + LE
7	2.66	467	0.009	H-9→L (0.58)	LMCT + LE
8	2.66	467	0.009	H-8→L (0.58)	LMCT + LE
17	2.98	416	0.022	H-15→L (0.59)	LMCT + LE
18	3.00	414	0.016	H-18→L (0.46)	LMCT + LE
19	3.00	414	0.016	H-19→L (0.46)	LMCT + LE
20	3.02	410	0.018	H-17→L (0.64)	LMCT + LE
39	3.31	374	0.028	H-23→L (0.25),H-3→L+2 (-0.22), H-4→L+1 (-0.22), H-13→L (0.22)	LMCT + LE
40	3.31	374	0.028	H-22→L (0.25), H-3→L+1 (0.22), H-4→L+2 (-0.22), H-14→L (0.22)	LMCT + LE
44	3.36	369	0.022	H-31→L (0.39)	LMCT + LE

Table S11. Selected excited states of Ag_{79} NC with transition energy (ΔE), wavelength (λ), oscillator strength (f) and characters. (H, HOMO; L, LUMO).

State	ΔE (eV)	λ (nm)	f	Character	
1	2.30	538	0.005	H→L (0.80)	LMCT + LE
2	2.38	521	0.008	H-1→L (0.82)	LMCT + LE
3	2.45	507	0.009	H-3→L (0.55), H-4→L (0.52)	LMCT + LE
4	2.48	499	0.008	H-3→L (0.58), H-4→L (-0.52)	LMCT + LE
15	2.89	428	0.024	H-11→L (0.45)	LMCT + LE
18	2.97	417	0.023	H-19→L (0.35)	LMCT + LE
20	2.99	414	0.025	H-16→L (0.53)	LMCT + LE
36	3.22	385	0.044	H-2→L+2 (0.30)	LMCT + LE
38	3.24	383	0.035	H-2→L+1 (0.25), H-20→L (-0.24)	LMCT + LE
46	3.33	372	0.023	H-26→L (0.30)	LMCT + LE
47	3.34	371	0.03	H-3→L+3 (0.29)	LMCT + LE
48	3.35	370	0.024	H-30→L (0.22), H-24→L (-0.21)	LMCT + LE
50	3.37	368	0.022	H-4→L+2 (0.27)	LMCT + LE

Table S12. Contributions of the atomic species and additive Ag and S atoms to MOs of Ag_{78} NC (H, HOMO; L, LUMO).

MO	Contributions of atomic species (%)		
	Ag	S	Others
H-31	39	48	13
H-23	39	51	11
H-22	39	51	11
H-19	37	55	8
H-18	37	55	8
H-17	36	56	8
H-15	35	56	8
H-14	36	56	8
H-13	36	56	8
H-10	35	60	6
H-9	37	57	6
H-8	37	57	6
H-4	36	56	7

H-3	36	56	7
H-1	37	58	5
L	77	17	6
L+1	71	22	6
L+2	71	22	6

Table S13. Contributions of the atomic species and additive Ag and S atoms to MOs of Ag_{79} NC (H, HOMO; L, LUMO).

MO	Contributions of atomic species (%)			Contribution of additive atoms (%)	
	Ag	S	Others	Ag	S
H-30	40	52	9	0.04	0.17
H-26	39	52	9	-0.03	0.91
H-24	37	54	9	-0.01	0.69
H-20	39	52	9	0.11	0.28
H-19	36	57	7	-0.08	1.28
H-16	35	58	7	0.15	1.64
H-11	36	57	7	0.28	2.87
H-4	36	60	4	0.11	3.98
H-3	37	59	4	0.26	3.21
H-2	37	60	4	0.23	4.43
H-1	37	60	3	0.05	0.12
H	37	60	3	0.03	1.89
L	78	14	7	4.28	0.17
L+1	75	18	7	4.31	0.13
L+2	75	19	7	8.97	0.15
L+3	77	16	7	3.32	0.29

Table S14. Radiative and noon-radiative parameters of emission.

Nanocluster	Φ	τ /ns	k_r /s^{-1}	k_{nr} /s^{-1}
Ag_{79}	0.0773	1144	6.759×10^4	8.064×10^5
Ag_{78}	0.0010	22.45	4.494×10^4	4.449×10^7

Table S15. Temperature dependence emission parameters.

Nanocluster	Temperature/K	PL Intensity/a. u.
Ag_{79}	293	822
	77	8231
Ag_{78}	293	7
	77	1563

Table S16. Emission lifetime for both NCs.

		Deoxygenated				Oxygenated		
Ag_{79}		τ / ns	α	f		τ / ns	α	f
	1	876.3	0.572	0.571	1	160.2	0.594	0.335
	2	299.5	0.268	0.092	2	396.7	0.364	0.507
	3	1848	0.160	0.337	3	1058	0.042	0.158
	τ_{av}	1144			τ_{av}	422.4		
Ag_{78}		τ / ns	α	f		τ / ns	α	f
	1	2.442	0.396	0.094	1	2.099	0.483	0.122
	2	9.171	0.470	0.417	2	9.448	0.404	0.459
	3	37.61	0.134	0.489	3	30.87	0.113	0.419
	τ_{av}	22.45			τ_{av}	17.54		

Table S17. Oxygen quenching coefficient and the Stern-Volmer coefficient of Ag_{78} and Ag_{79} NCs.

Nanocluster	k_q / M^{-1}s^{-1}	K_{SV} / M^{-1}
Ag_{79}	1.400	161.1
Ag_{78}	1.165×10^4	26.16×10^4

Table S18. Total energy at the T_1-state geometry, emission energy (ΔE), and wavelength (λ).

	S_0 (au)	T_1 (au)	ΔE (eV)	λ (nm)
Ag_{78}	-43516.12481	-43516.07223	1.431	867
Ag_{79}	-45858.18176	-45858.14160	1.093	1135

Table S19. Comparison of PL emission QY with the reported literature for high nuclear metal NCs and nanoparticles

Nanocluster	PL QY (%)	Reference
$[Ag_{27}(S^{t}Bu)_{14}(S)_2(CF_3COO)_9(DMAc)_4]\cdot DMAc$	0	S19
$[Ag_{28}(AdmS)_{14}(S)_2(CF_3COO)_{10}(H_2O)_4]$	3.97	S19
$[Ag_{29}(BDT)_{12}]^{3-}$	0.45	S20
$[Ag_{29}(BDT)_{12}(PPh3)_4]^{3-}$	0.9	S20
$[Ag_{29}(BDT)_{12}(DPPM)_4]^{3-}$	2.9	S20
$[Ag_{29}(BDT)_{12}(DPPE)_4]^{3-}$	6.24	S20
$[Ag_{62}S_{13}(SBu^{t})_{32}](BF_4)_4$	0.014	S21
$[Ag_{62}S_{12}(SBu^{t})_{32}]^{2+}$	0	S22
$[Ag_{50}S_{13}(S^{t}Bu)_{20}][CF_3COO]_4$	0.82	S23
$[Cu_{47}(PhSe)_{15}(PPh_3)_5(CF_3COO)_{12}H_{12}]$	2.74	S24
$[(CuAg)_{47}(PhSe)_{18}(PPh_3)_6(CF_3COO)_{12}H_6]^{3+}$	0.31	S24
$Au_{52}(SC_4H_4C(CH_3)_3)$	3.8	S25
Ag_2S	0.2	S26
Surface modified Ag_2S	2.3	S26
Ag_{78}	0.1	This work
Ag_{79}	7.73	This work

$S^{t}Bu$: tert-butyl thiolate; AdmS: adamantanethiolate; DPPM: 1,1-bis(diphenylphosphino)methane; DPPE: 1,2-bis(diphenylphosphino)ethane; BDT: 1,3-Benzenedithiolate;

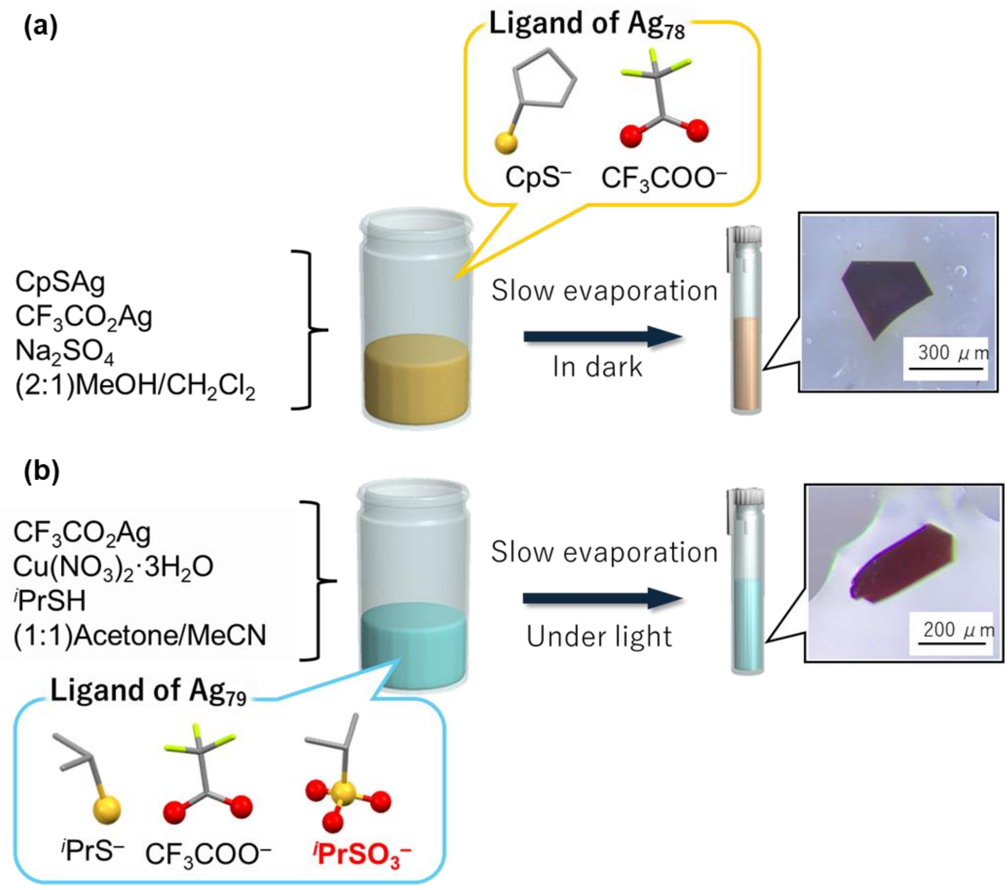

Figure S1. Schematic representation of the reactions and Optical microscope image of (a) Ag_{78} NC and (b) Ag_{79} NC.

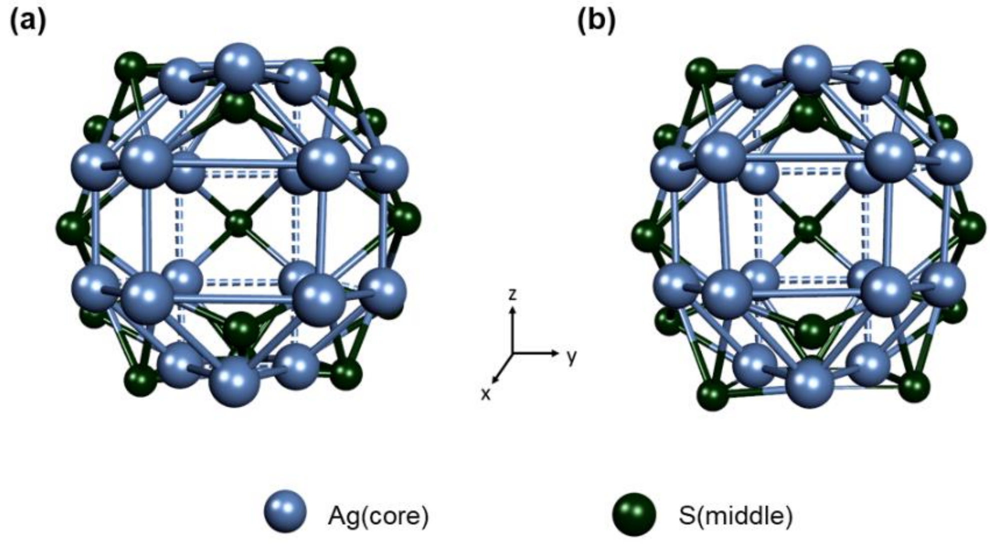

Figure S2. Position of the $[S]^{2-}$ ligands (a) Ag_{78} NC and (b) Ag_{79} NC.

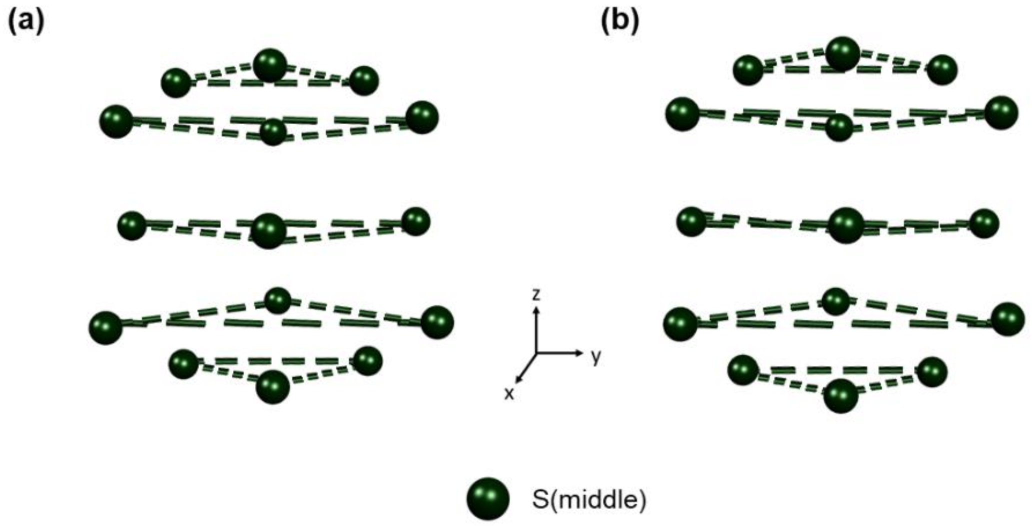

Figure S3. Five-layer distribution of $[S]^{2-}$ ligands (a) Ag_{78} NC and (b) Ag_{79} NC.

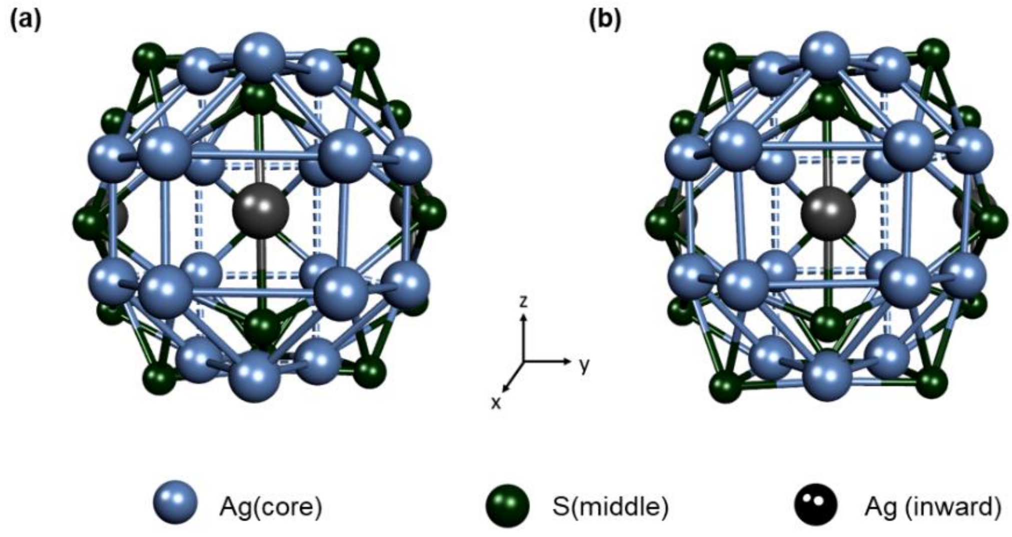

Figure S4. Position of the inward Ag atoms of outer shell in (a) Ag_{78} NC and (b) Ag_{79} NC.

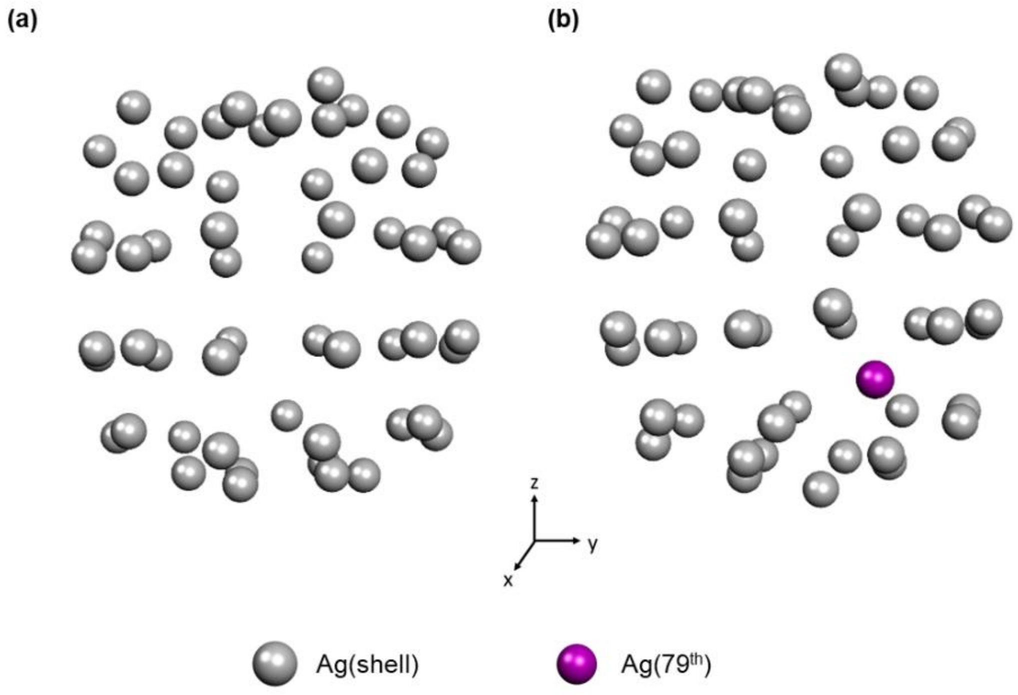

Figure S5. Arrangement of outer most Ag atoms in (a) Ag_{78} NC and (b) Ag_{79} NC.

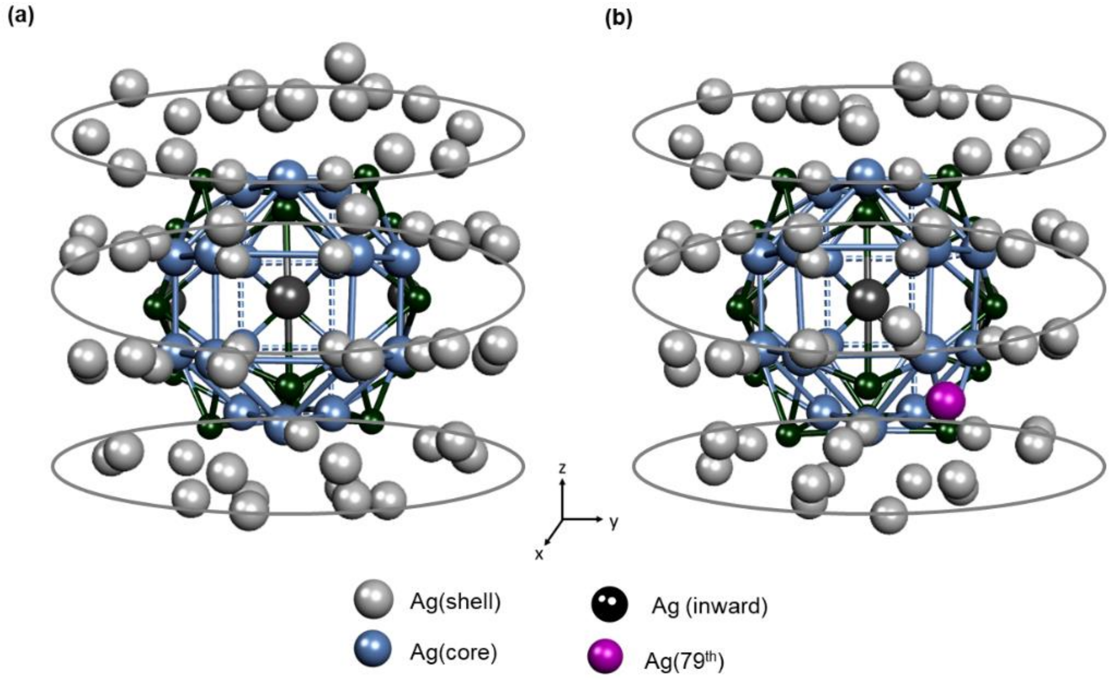

Figure S6. Other layer of the outermost cationic shell contains (a) 57 Ag(I) atoms for Ag_{78} NC and (b) 58 Ag(I) atoms for Ag_{79} NC.

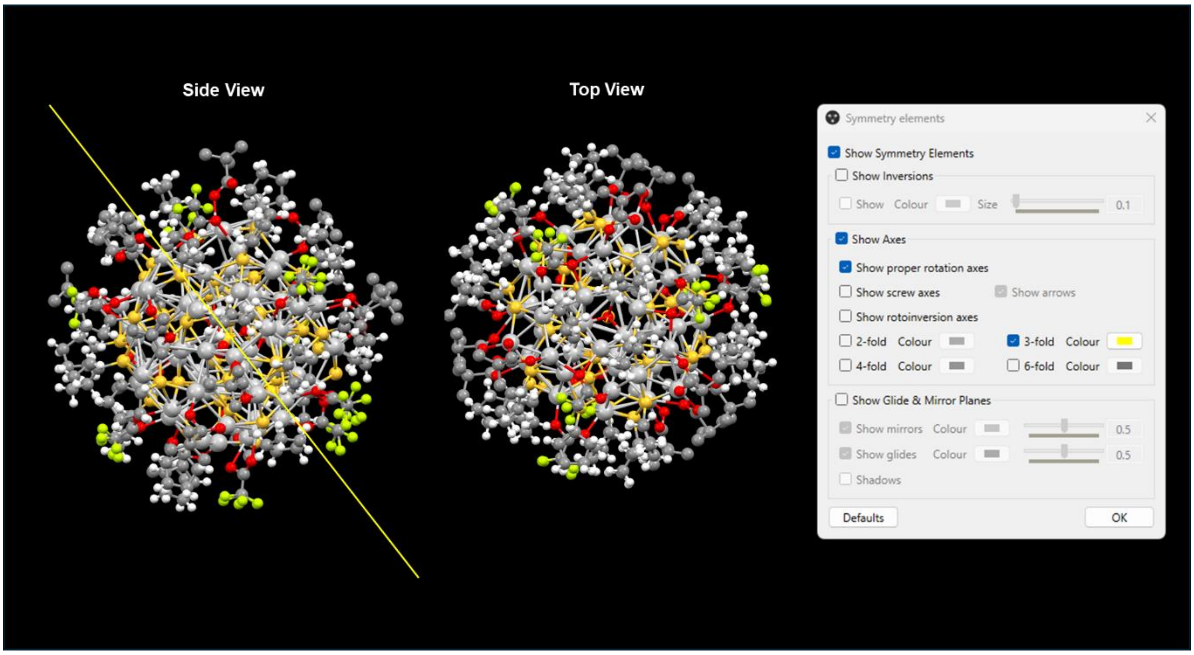

Figure S7. The C_3 axis is shown passing through one of the oxygen atoms of the $[SO_4]^{2-}$ anion and its sulphur atom, which is located at the centre of the NC. The free version, Free Mercury, obtained through the CSD-Community software suite was used for showing C_3 axis**.**

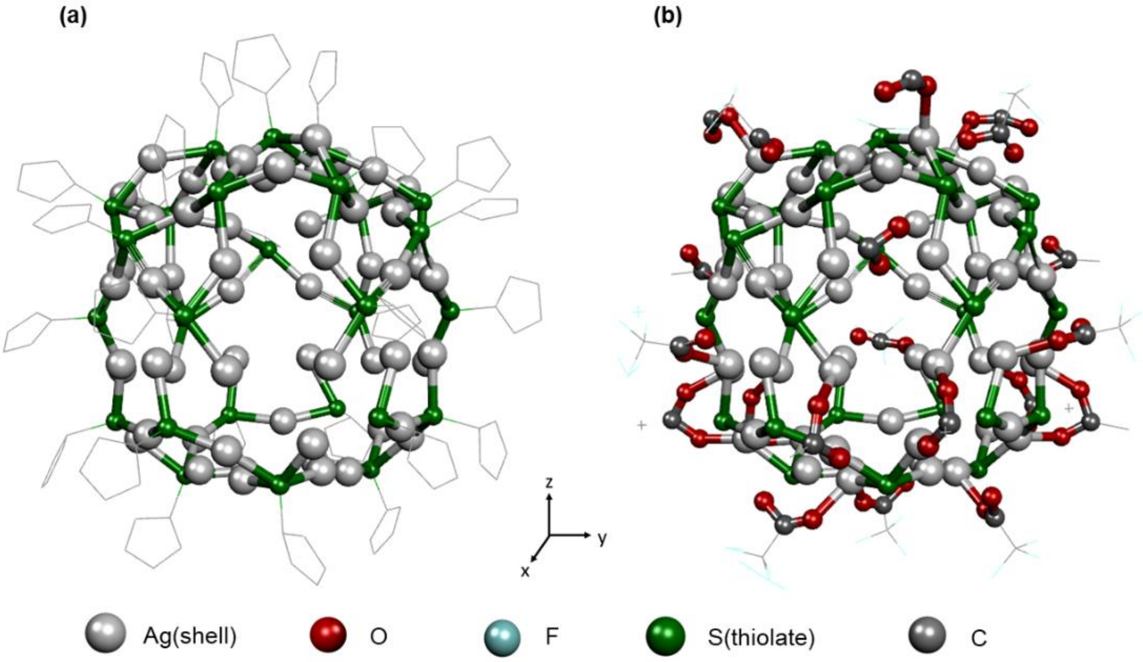

Figure S8. Ag_{78} NC (a) outer most layer with CpS^- coordinations, (b) $CF_3CO_2^-$ coordination.

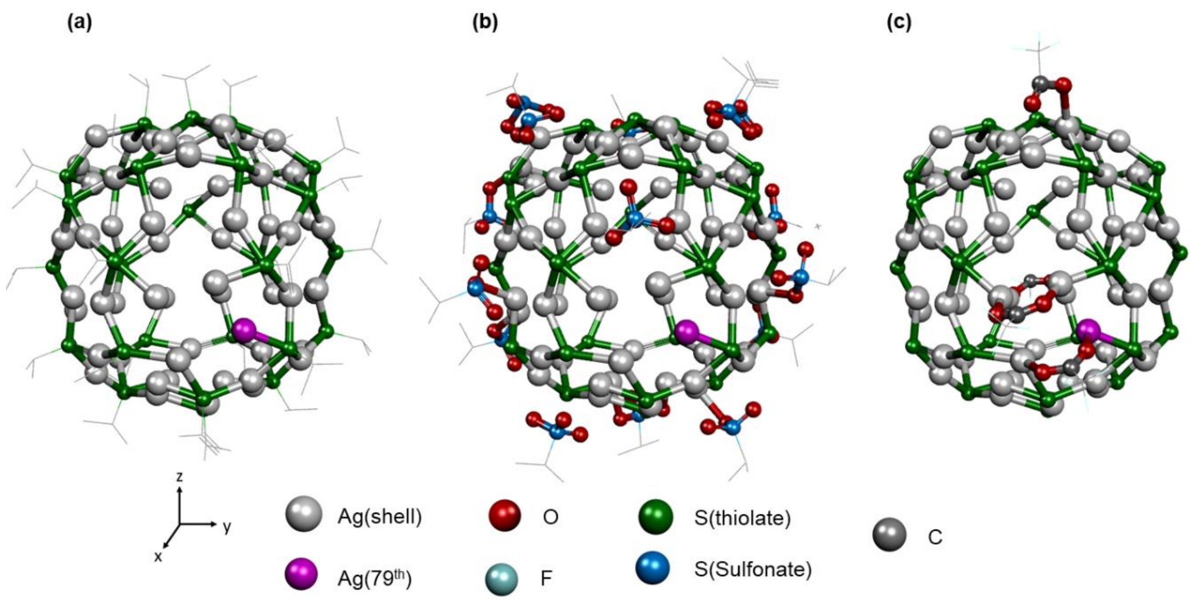

Figure S9. Ag_{79} NC (a) ^{i}PrS (b) $^{i}PrSO_3^-$ (c) $CF_3CO_2^-$ coordination.

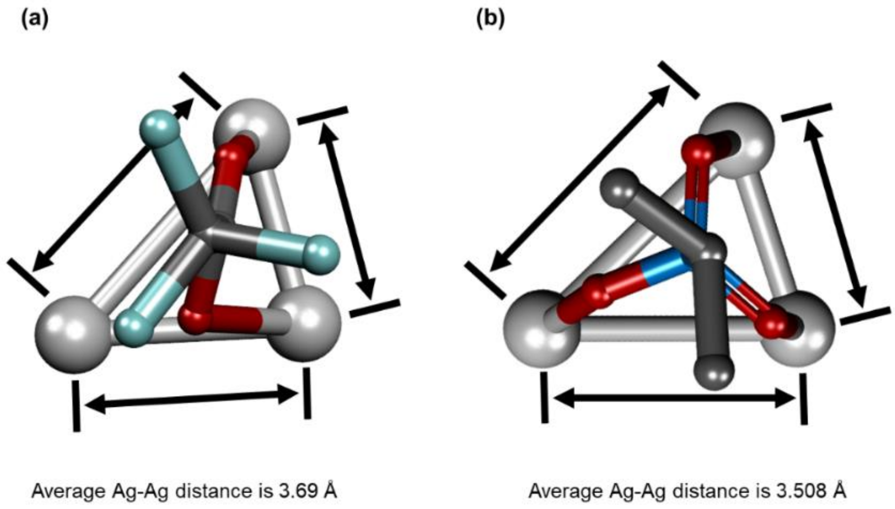

Figure 10. Average Ag-Ag bond length at the coordinating sites (a) Ag_{78} NC with bidentate ligand coordination and (b) Ag_{79} NC with tridentate ligand coordination.

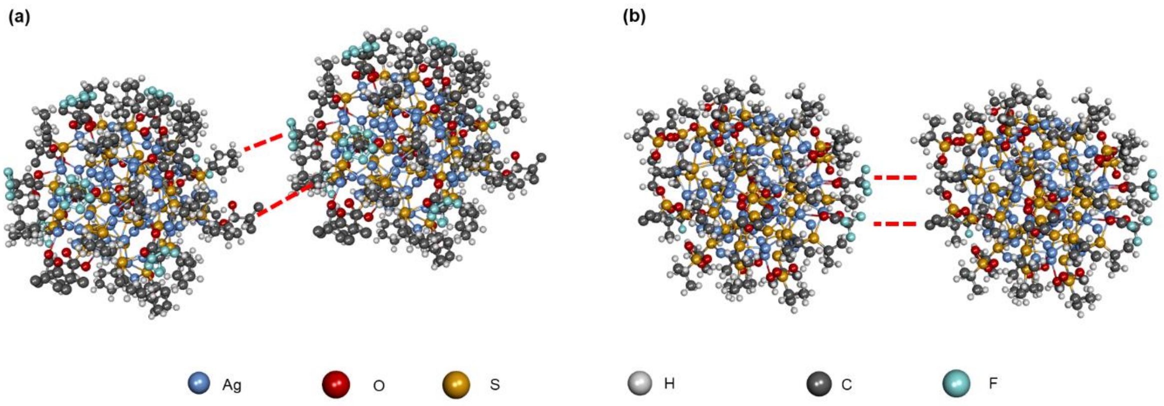

Figure S11. Inter cluster interactions in (a) Ag_{78} (b) Ag_{79} NCs.

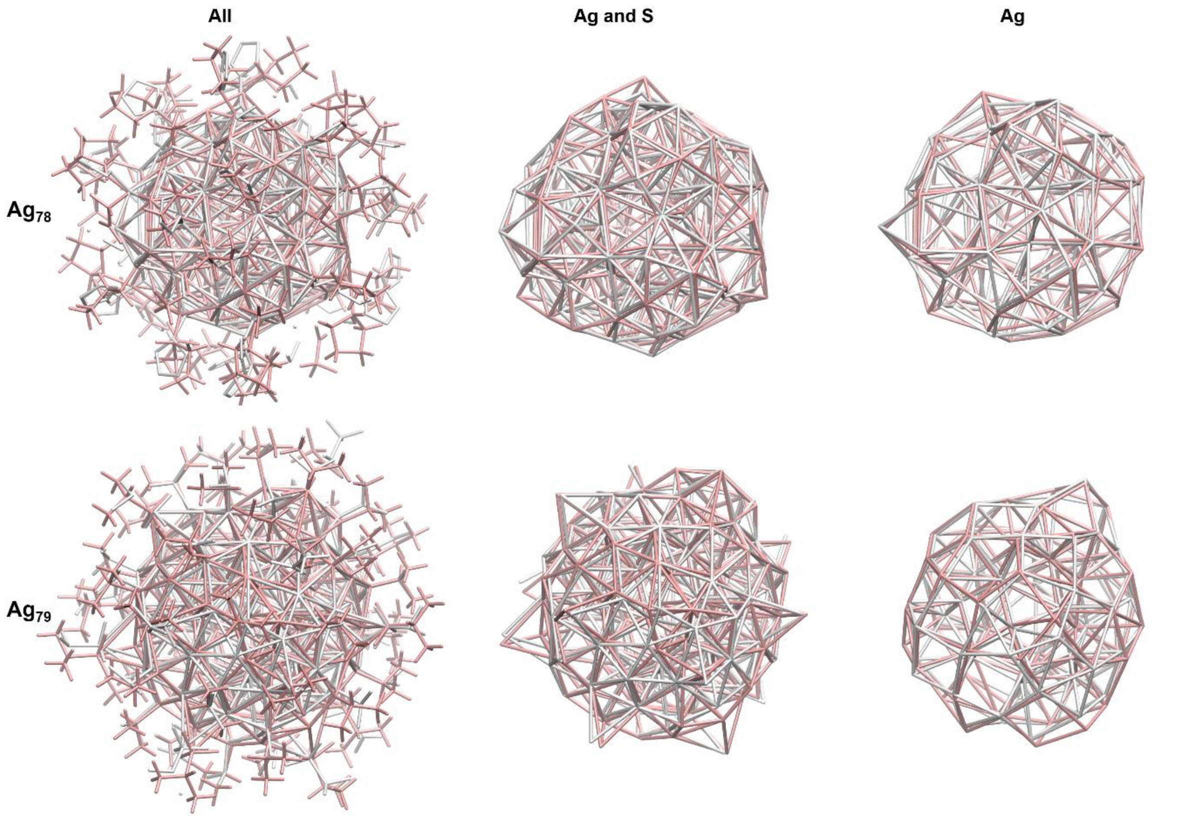

Figure S12. Comparison of the structures of Ag_{78} and Ag_{79} NC. The SCXRD structures (white) and DFT calculations (pink) of the ground states. The superpositions of the structures were calculated based on the Kabsch algorithm.

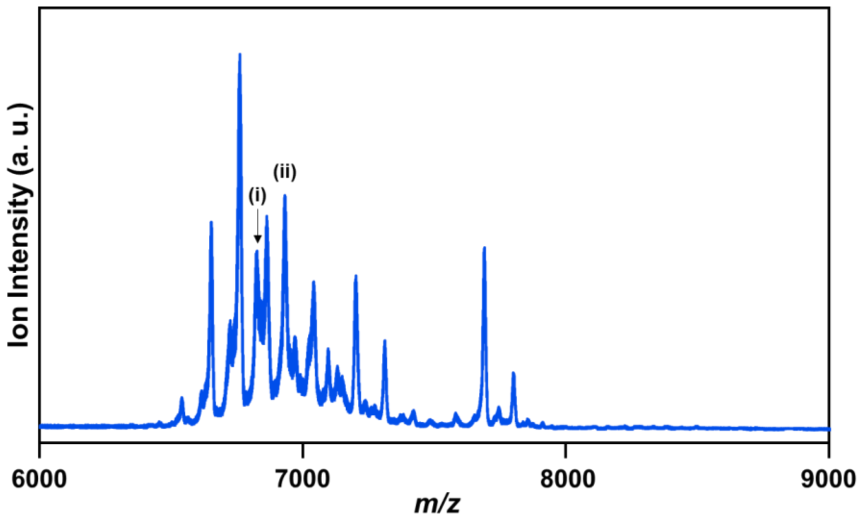

Figure S13. ESI-MS of Ag_{78} NC.

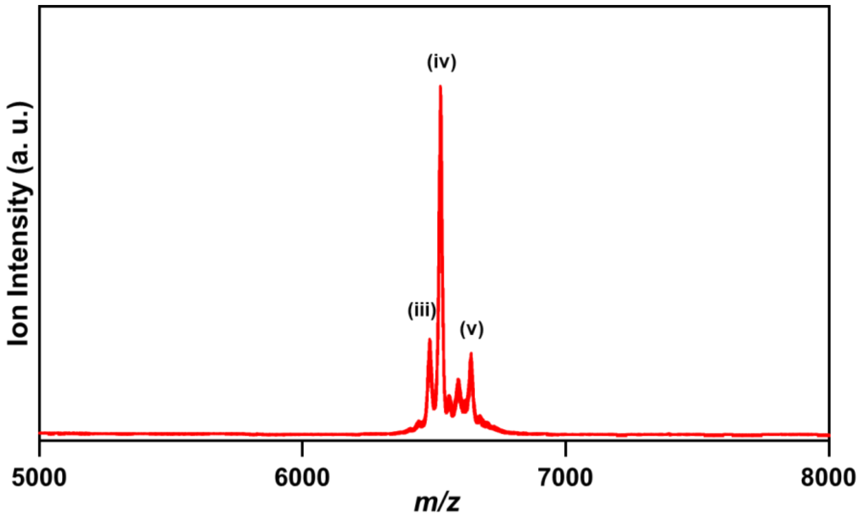

Figure S14. ESI-MS Ag_{79} NC.

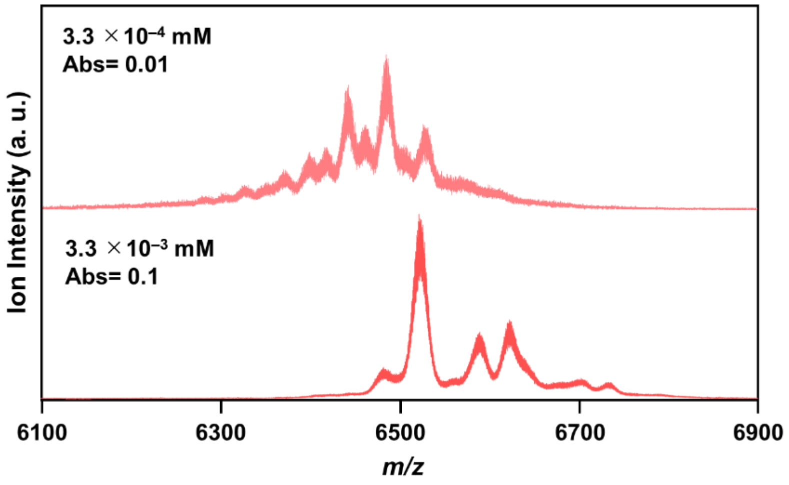

Figure S15. ESI-MS of Ag_{79} NC with different concentrations.

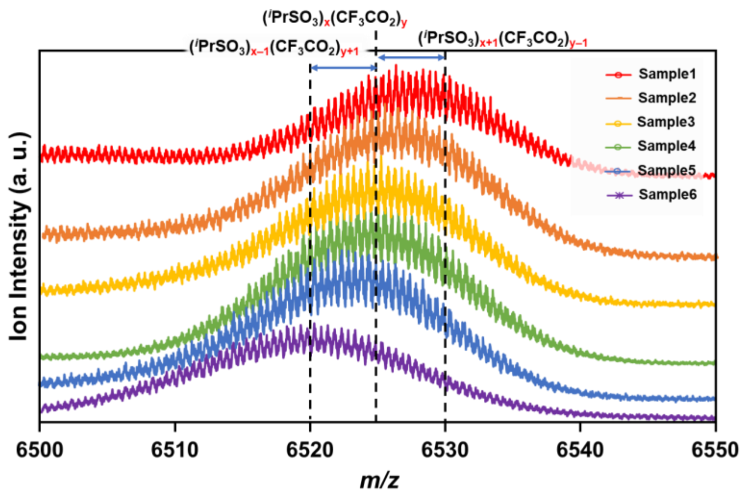

Figure S16. Shifting of the equilibrium in ESI-MS of Ag_{79} NC with different concentrations.

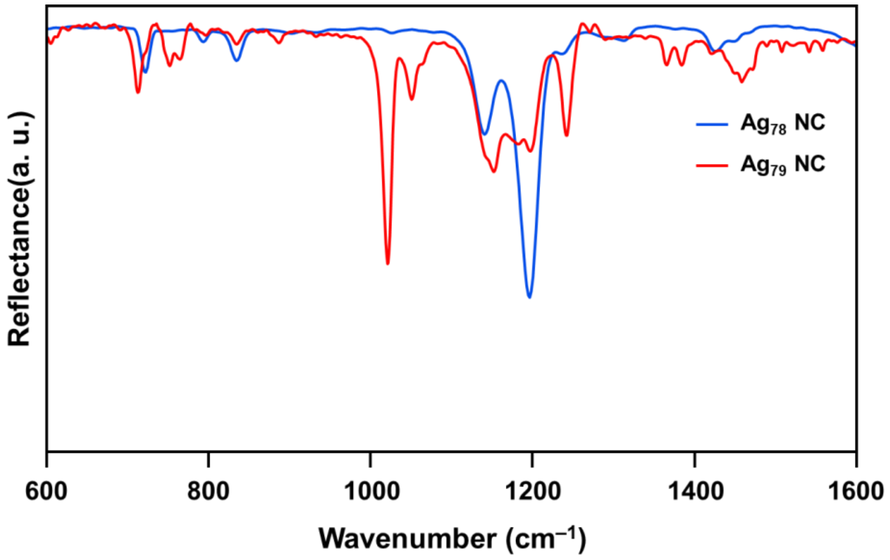

Figure S17. FT-IR spectra of both Ag_{78} and Ag_{79} NCs.

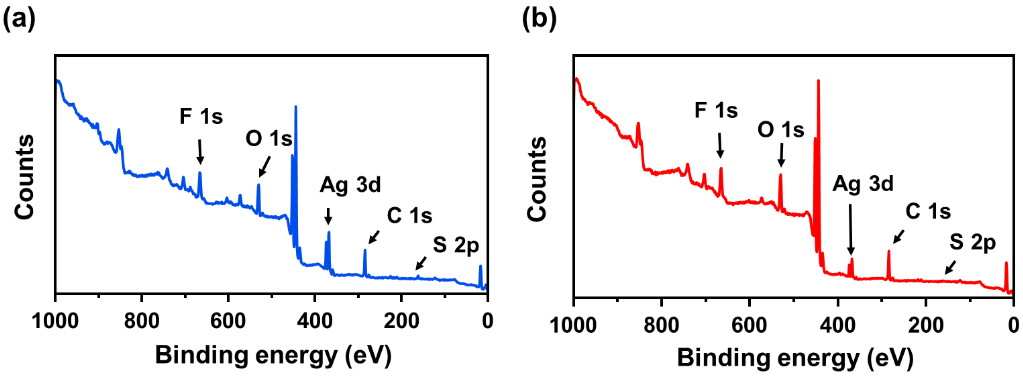

Figure S18. XPS survey spectrum for both (a) Ag_{78} and (b) Ag_{79} NCs.

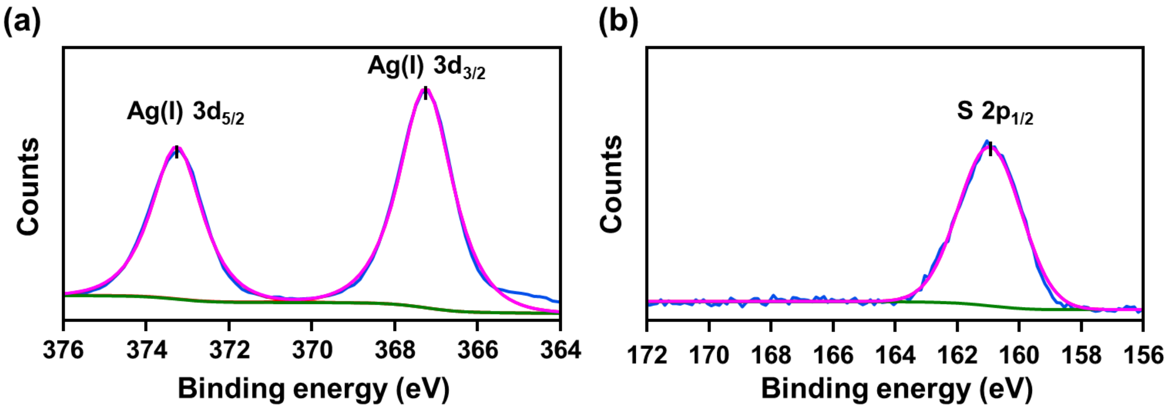

Figure S19. Deconvoluted XPS data of Ag_{78} NC (a) Ag 3d and (b) S 2p.

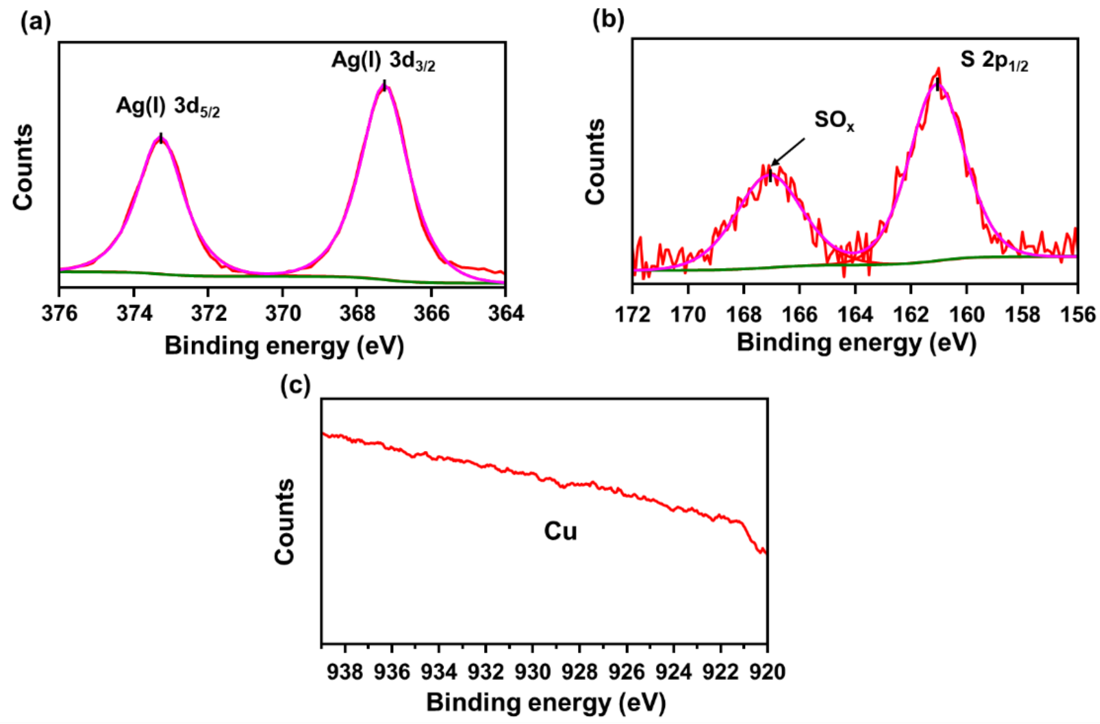

Figure S20. Deconvoluted XPS data of Ag_{79} NC (a) Ag 3d, (b) S 2p and (c) the binding energy region of Cu (no peak at this region confirms the absence of Cu atom in the NC structure although we have added the Cu precursor salt during the reaction).

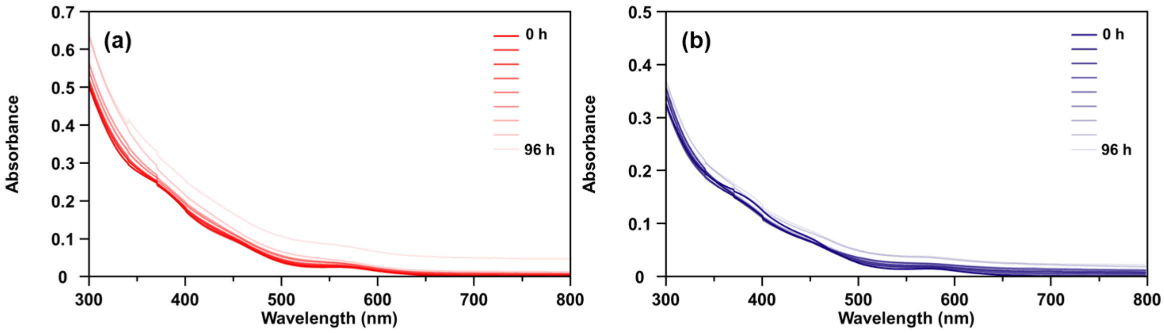

Figure S21. Time dependent UV-vis spectra of (a) Ag_{78} (b) Ag_{79} NCs in solution.

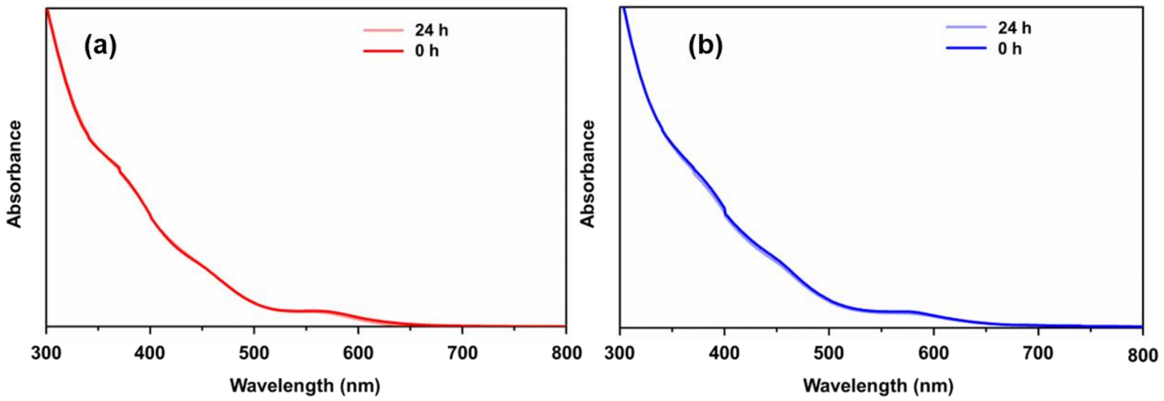

Figure S22. Photostability of (a) Ag_{78} (b) Ag_{79} NCs in solution under ambient light condition.

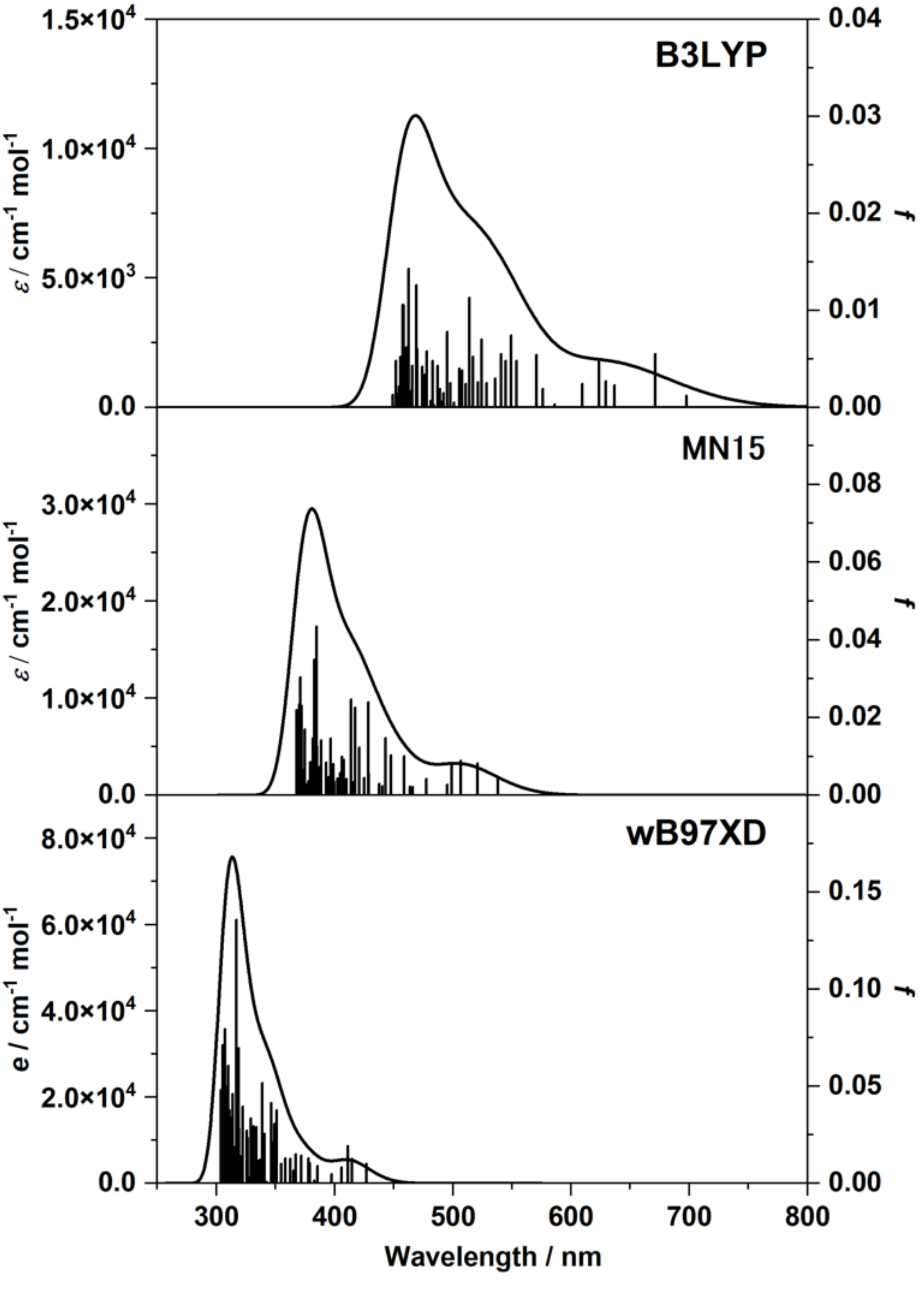

Figure S23. DFT functional dependence of the absorption spectrum of Ag_{79} NC. Each peak was convoluted by Gaussian functions with half the bandwidth at 1/e peak height of 0.15 eV.

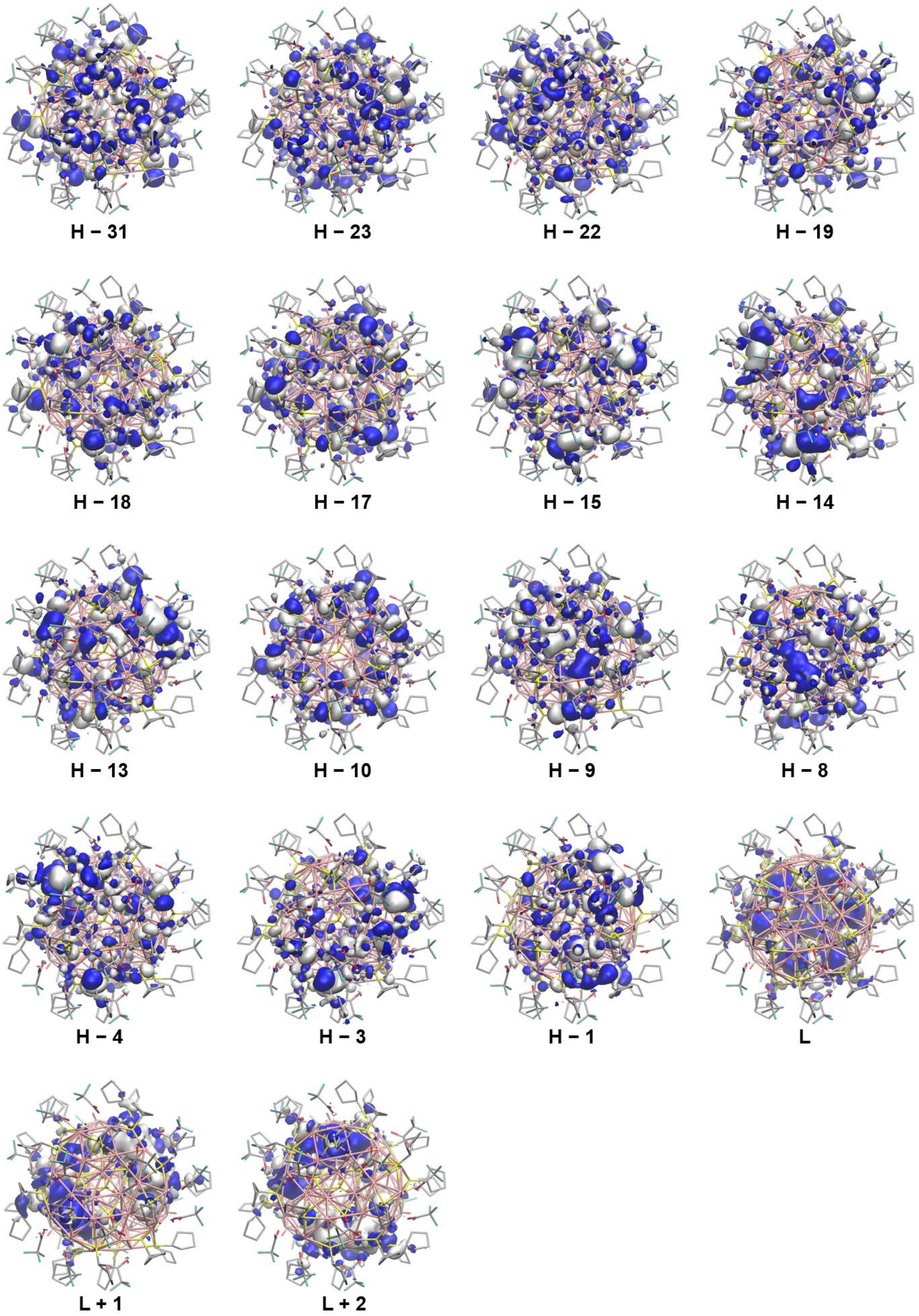

Figure S24. MOs relevant to the absorption spectrum of Ag_{78} NC. H, HOMO; L, LUMO. The iso-surface value is |0.01| a.u.

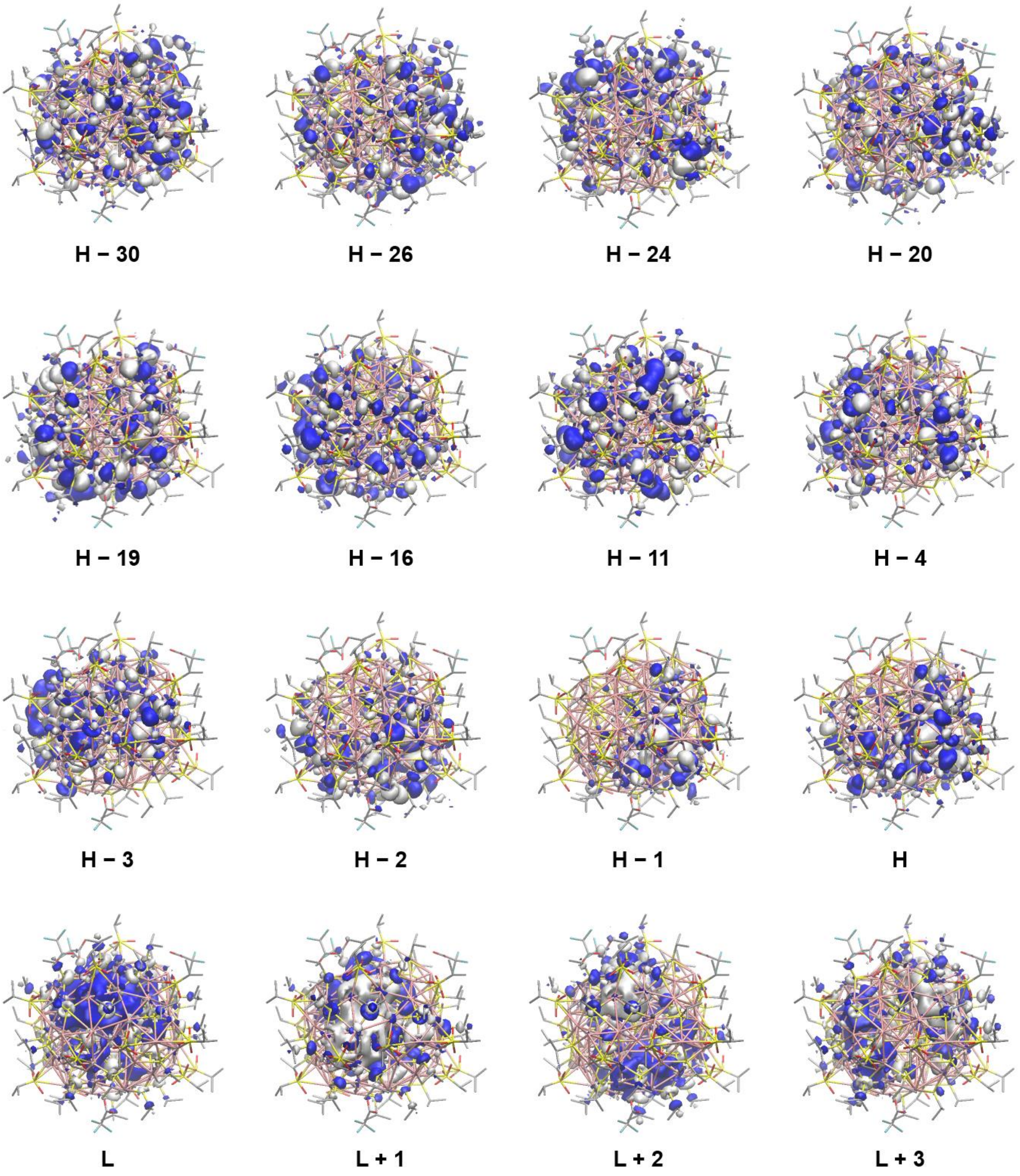

Figure S25. MOs relevant to the absorption spectrum of Ag_{79} NC. H, HOMO; L, LUMO. The iso-surface value is |0.01| a.u.

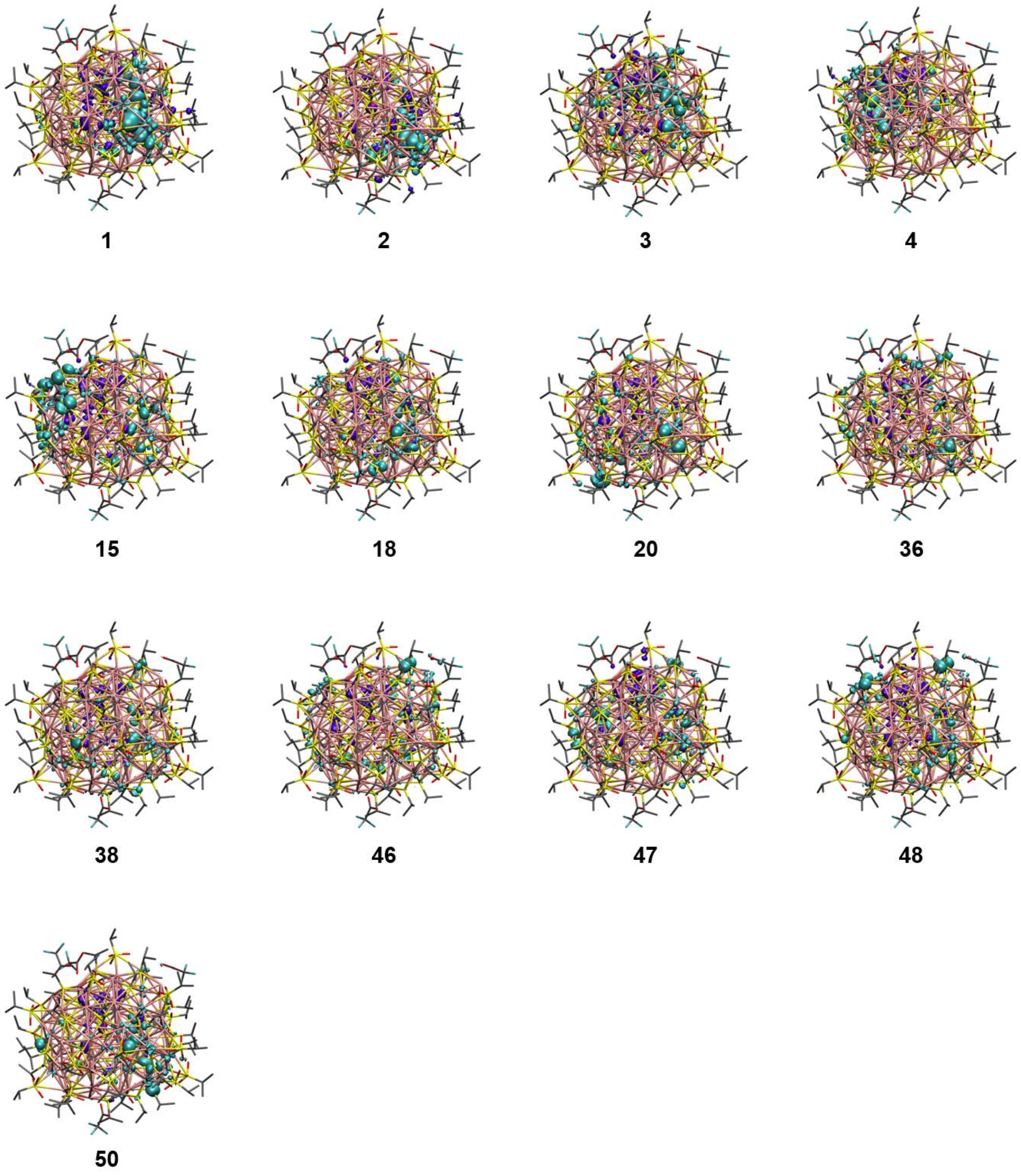

Figure S26. Electron density differences between ground and excited states of Ag_{79} NC. The numbers below the structures are those of the excited states. The increment and decrement of the electron density are represented by orange and cyan, respectively. The iso-surface value is |0.0005| a.u.

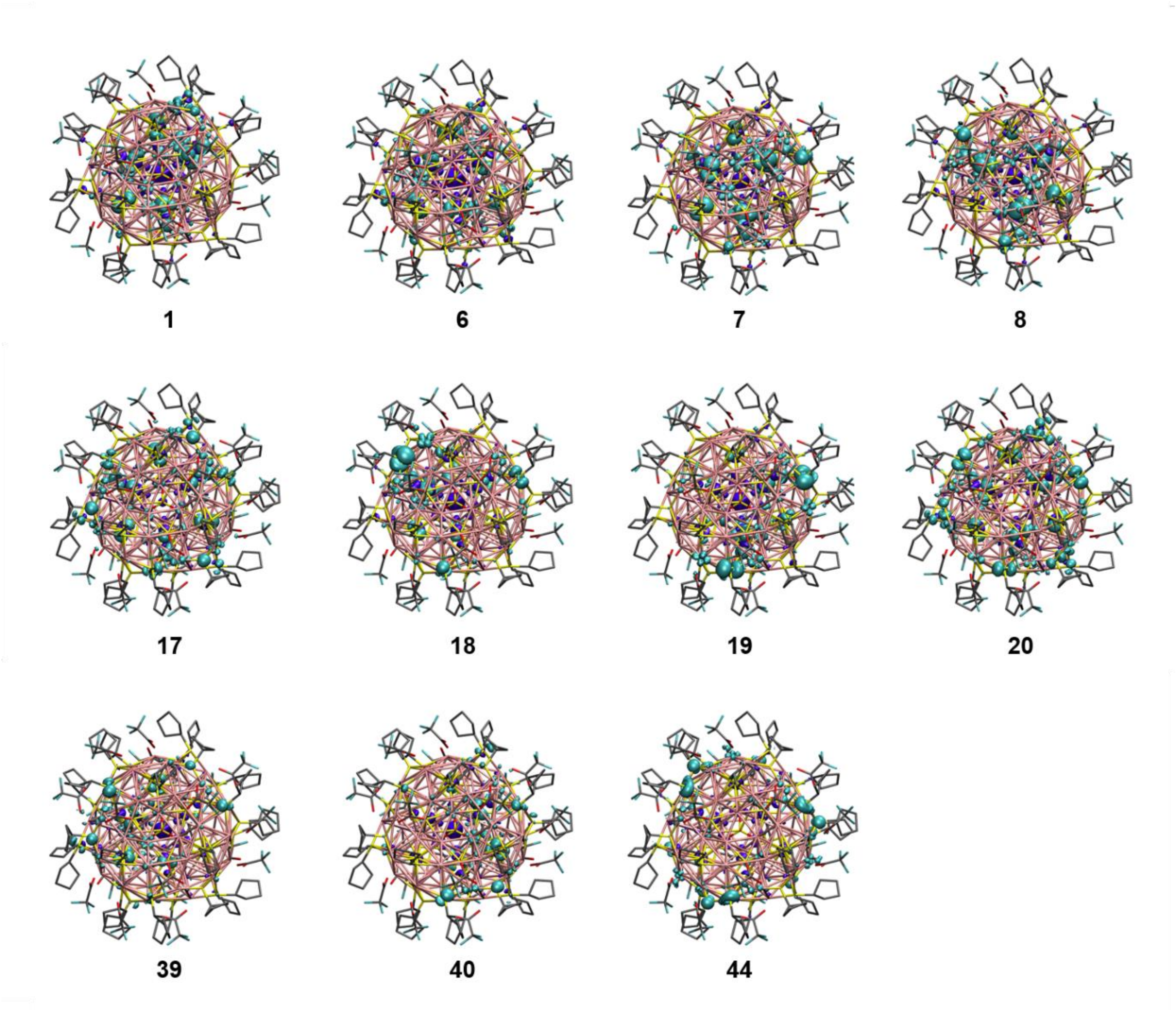

Figure S27. Electron density differences between ground and excited states of Ag_{78} NC. The numbers below the structures are those of the excited states. The increment and decrement of the electron density are represented by orange and cyan, respectively. The iso-surface value is |0.0005| a.u.

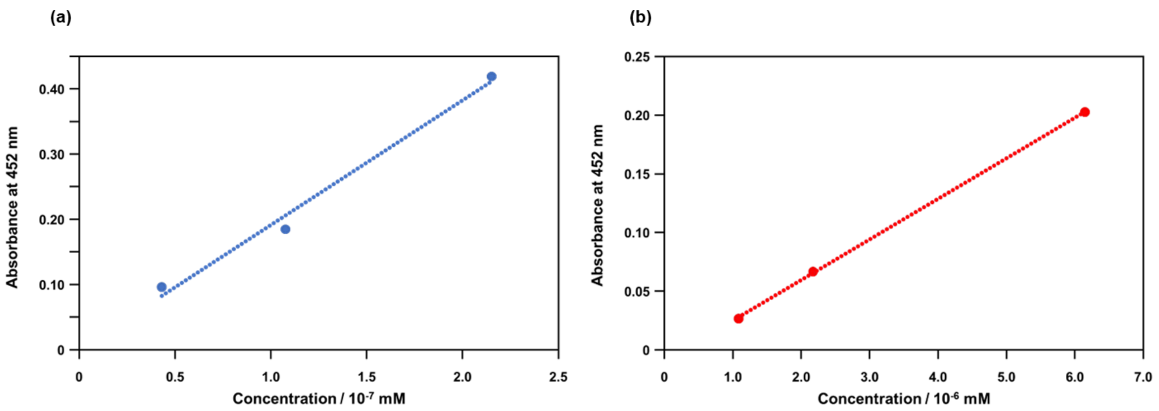

Figure S28. Molar absorption coefficient of (a) Ag_{78} NC and (b) Ag_{79} NC in dichloromethane at 452 nm.

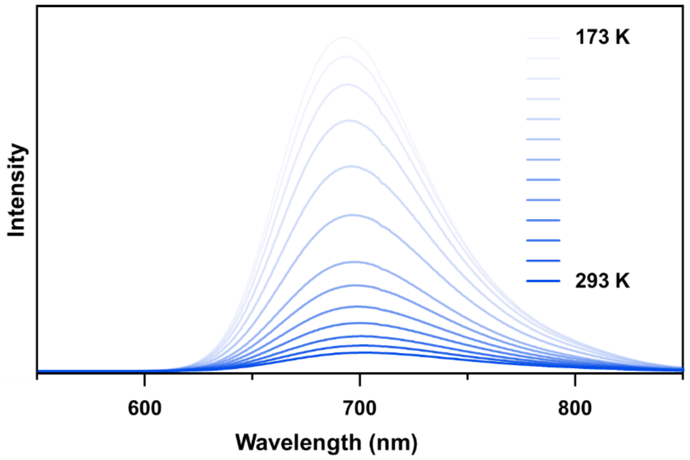

Figure S29. Temperature dependent PL spectra of Ag_{79} NC.

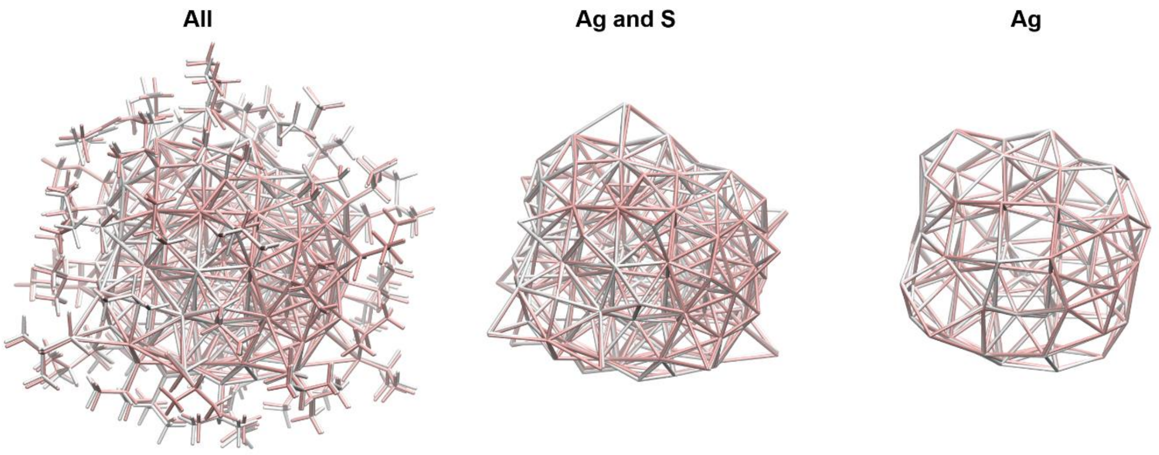

Figure S30. Comparison of the structures of Ag_{79} NC in the singlet (white) and triplet (pink) states. The superpositions of the structures were calculated based on the Kabsch algorithm.

References

S1. Cheng, L.-P.; Wang, Z.; Wu, Q.-Y.; Su, H.-F.; Peng, T.; Luo, G.-G.; Li, Y.-A.; Sun, D.; Zheng, L.-S. Small Size Yet Big Action: a Simple Sulfate Anion Templated a Discrete 78-nuclearity Silver Sulfur Nanocluster with a Multishell Structure. *Chem. Commun.* **2018**, *54*, 2361-2364

S2. Wagner, C. D. NIST X-ray Photoelectron Spectroscopy Database. *NIST Standarad Reference Database 20* **2000**.

S3. Valeur, B.; Berberan-Santos, M. N. *Molecular Fluorescence: Principles and Applications*; John Wiley & Sons, **2013**.

S4. Rurack, K.; Spieles, M. Fluorescence Quantum Yields of a Series of Red and Near-Infrared Dyes Emitting at 600− 1000 nm. *Anal. Chem.* **2011**, *83*, 1232-1242.

S5. Sheldrick, G. Crystal Structure Refinement with SHELXL. *Acta Crystallogr., Sect. C: Struct. Chem.* **2015**, *71*, 3-8.

S6. Bruker APEX3, v2019.1–0, Bruker AXS Inc., Madison, WI, USA, **2019**.

S7. Dolomanov, O. V.; Bourhis, L. J.; Gildea, R. J.; Howard, J. A.; Puschmann, H. OLEX2: a Complete Structure Solution, Refinement and Analysis Program. *J. Appl. Crystallogr.* **2009**, *42*, 339-341.

S8. Horita, Y.; Hossain, S.; Ishimi, M.; Zhao, P.; Sera, M.; Kawawaki, T.; Takano, S.; Niihori, Y.; Nakamura, T.; Tsukuda, T.; Ehara, M.; Negishi, Y. Clarifying the Electronic Structure of Anion-Templated Silver Nanoclusters by Optical Absorption Spectroscopy and Theoretical Calculation. *J. Am. Chem. Soc.* **2023**, *145*, 23533-23540.

S9. Yu, H. S.; He, X.; Li, S. L.; Truhlar, D. G. MN15: A Kohn–Sham Global-Hybrid Exchange–Correlation Density Functional with Broad Accuracy for Multi-Reference and Single-Reference Systems and Noncovalent Interactions. *Chem. Sci.* **2016**, *7*, 5032-5051.

S10. Binkley, J. S.; Pople, J. A.; Hehre, W. J. Self-consistent Molecular Orbital Methods. 21. Small Split-Valence Basis Sets for First-Row Elements. *J. Am. Chem. Soc.* **1980**, *102*, 939-947.

S11. Hay, P. J.; Wadt, W. R. Ab Initio Effective Core Potentials for Molecular Calculations. Potentials for the Transition Metal Atoms Sc to Hg. *J. Chem. Phys.* **1985**, *82*, 270-283.

S12. Frisch, M. J.; Trucks, G. W.; Schlegel, H. B.; Scuseria, G. E.; Robb, M. A.; Cheeseman, J. R.; Scalmani, G.; Barone, V.; Petersson, G. A.; Nakatsuji, H. et al, *Gaussian 16 rev. C02*, Wallingford, CT, 2016.

S13. Foster, J. P.; Weinhold, F. Natural Hybrid Orbitals. *J. Am. Chem. Soc.* **1980**, *102*, 7211-7218.

S14. Naaman, R.; Vager, Z. *The Structure of Small Molecules and Ions*; Springer Science & Business Media, **2012**.

S15. O'boyle, N. M.; Tenderholt, A. L.; Langner, K. M. Cclib: a Library for Package-Independent computational chemistry algorithms. *J. Comput. Chem.* **2008**, *29* (5), 839-845.

S16. Lu, T.; Chen, F. Multiwfn: A Multifunctional Wavefunction Analyzer. *J. Comput. Chem.* **2012**, *33*, 580-592.

S17. Kabsch, W. A Solution for the Best Rotation to Relate two Sets of Vectors. *Found. Crystallogr.* **1976**, *32* (5), 922-923.

S18. Calculate Root-mean-square deviation (RMSD) of Two Molecules Using Rotation, GitHub, http://github.com/charnley/rmsd.

S19. Yang, J.-S.; Han, Z.; Dong, X.-Y.; Luo, P.; Mo, H.-L.; Zang, S.-Q. Extra Silver Atom Triggers Room-Temperature Photoluminescence in Atomically Precise Radarlike Silver Clusters. *Angew. Chem. Int. Ed.* 2020, *59*, 11898-11902.

S20. Khatun, E.; Ghosh, A.; Chakraborty, P.; Singh, P.; Bodiuzzaman, M.; Ganesan, P.; Nataranjan, G.; Ghosh, J.; Pal, S. K.; Pradeep, T. A Thirty-fold Photoluminescence Enhancement Induced by Secondary Ligands in Monolayer Protected Silver Clusters. *Nanoscale* 2018, *10*, 20033-20042.

S21. Li, G.; Lei, Z.; Wang, Q.-M. Luminescent Molecular Ag–S Nanocluster $[Ag_{62}S_{13}(SBu^t)_{32}](BF_4)_4$. *J. Am. Chem. Soc.* 2010, *132*, 17678-17679.

S22. Jin, S.; Wang, S.; Song, Y.; Zhou, M.; Zhong, J.; Zhang, J.; Xia, A.; Pei, Y.; Chen, M.; Li, P.; et al. Crystal Structure and Optical Properties of the $[Ag_{62}S_{12}(SBu^t)_{32}]^{2+}$ Nanocluster with a Complete Face-Centered Cubic Kernel. *J. Am. Chem. Soc.* 2014, *136*, 15559-15565.

S23. Biswas, S.; Das, A. K.; Reber, A. C.; Biswas, S.; Bhandary, S.; Kamble, V. B.; Khanna, S. N.; Mandal, S. The New Ag–S Cluster $[Ag_{50}S_{13}(S^tBu)_{20}][CF_3COO]_4$ with a Unique hcp Ag_{14} Kernel and Ag_{36} Keplerian-Shell-Based Structural Architecture and Its Photoresponsivity. *Nano Lett.* 2022, *22*, 3721-3727.

S24. Sun, J.; Liu, J.; Su, H.-F.; Li, S.; Tang, X.; Xie, Z.; Xu, Z.; Jiang, W.; Wei, J.; Gong, X.; et al. Eight-electron copper-hydride nanoclusters: synthesis, structure, alloying chemistry and photoluminescence. *Chemical Science* 2025, *16* (15), 6392-6401.

S25. Wang, Y.; Liu, Z.; Mazumder, A.; Gianopoulos, C. G.; Kirschbaum, K.; Peteanu, L. A.; Jin, R. Tailoring Carbon Tails of Ligands on $Au_{52}(SR)_{32}$ Nanoclusters Enhances the Near-Infrared Photoluminescence Quantum Yield from 3.8 to 18.3%. *J. Am. Chem. Soc.* 2023, *145*, 26328-26338.

S26. Ortega-Rodríguez, A.; Shen, Y.; Zabala Gutierrez, I.; Santos, H. D. A.; Torres Vera, V.; Ximendes, E.; Villaverde, G.; Lifante, J.; Gerke, C.; Fernández, N.; et al. 10-Fold Quantum Yield Improvement of Ag_2S Nanoparticles by Fine Compositional Tuning. *ACS Appl. Mater. Interfaces* 2020, *12*, 12500-12509.
